# INDETERMINATE DOMAIN–DELLA protein interactions orchestrate gibberellin-mediated cell elongation in wheat and barley

**DOI:** 10.1073/pnas.2528934123

**Published:** 2026-01-30

**Authors:** Patrycja Sokolowska, Matthias Jöst, Wolfram Buss, Brett Ford, Peter Michael Chandler, Wolfgang Spielmeyer, Andrew L. Phillips, Alison K. Huttly, Danuše Tarkowská, Rocío Alarcón-Reverte, Suzanne J. Clark, Stephen Pearce, Peter Hedden, Stephen G. Thomas

**Affiliations:** ^a^Rothamsted Research, Harpenden AL5 2JQ, Hertfordshire, United Kingdom; ^b^Commonwealth Scientific and Industrial Research Organisation, Agriculture and Food, Canberra, ACT 2601, Australia; ^c^Laboratory of Growth Regulators, Institute of Experimental Botany, Czech Academy of Sciences and Palacky University, Olomouc CZ-77900, Czech Republic

**Keywords:** wheat, gibberellin, Rht1, semi-dwarf, IDD

## Abstract

DELLA proteins, members of the GRAS-domain family of transcriptional regulators, play a crucial role in plant growth and development. They modulate transcription indirectly via interactions with hundreds of transcription factors. The phytohormone gibberellin (GA) triggers DELLA degradation, providing a mechanism by which plants can integrate developmental and environmental signals to regulate gene expression and optimize growth responses. In agriculture, DELLA mutations have been instrumental in improving crop performance. Most modern wheat (*Triticum aestivum* L.) varieties carry *Rht-B1b* or *Rht-D1b* alleles that encode DELLA proteins resistant to GA-mediated degradation, resulting in constitutive partial suppression of stem growth, a semi-dwarf stature, and lodging resistance. However, these alleles also reduce early vigor and nitrogen use efficiency, limiting their utility in some environments. Understanding how DELLA proteins regulate growth and development is, therefore, critical for refining breeding strategies. In this study, we identified the orthologous C2H2 zinc-finger transcription factors *INDETERMINATE DOMAIN 5* (*IDD5*) in wheat and *SEMI-DWARF 3* (*SDW3*) in barley (*Hordeum vulgare*) as positive regulators of stem and leaf expansion. Both IDD5 and SDW3 physically interact with, and act downstream of, DELLA proteins as key components of GA-mediated growth responses. Altered expression levels of GA biosynthesis genes suggest that IDD5 contributes to GA homeostasis in addition to growth regulation. Loss-of-function mutations in *IDD5* and *SDW3* confer a GA-insensitive semi-dwarf phenotype comparable in height to the *Rht-D1b* Green Revolution allele. In field trials, *idd5* lines exhibited improved grain weight per spike but were lower-yielding due to reduced spike number.

## Significance

We identified orthologous cereal IDD transcription factors (IDD5 in wheat and SDW3 in barley) that interact with DELLA proteins to regulate plant height. By showing that IDD proteins promote stem and leaf expansion, our study opens avenues for fundamental research to understand growth pathways in cereals. We hypothesized that *idd5* loss-of-function mutations would mitigate some of the drawbacks of the Green Revolution semi-dwarfing alleles, but mutant lines were lower-yielding due to a reduction in spike number. Our study demonstrates the importance of replicated field trials to assess the potential agronomic benefits of novel genetic variants.

In higher plants, DELLA proteins are crucial regulators of various developmental processes, including cell elongation, flowering, and germination, as well as responses to both biotic and abiotic stresses (1). DELLA proteins regulate these processes via transcriptional reprogramming through interactions with members of at least 15 different families of transcription factors (TFs) (2, 3). These protein–protein interactions involve conserved motifs within the C-terminal GRAS domain of DELLA proteins (3–5).

In some cases, interaction with DELLA proteins inhibits the DNA-binding activity of target TFs to suppress transcriptional activity. For example, DELLA proteins bind to and sequester PHYTOCHROME INTERACTING FACTOR 4 (PIF4) which promotes cell elongation, and GROWTH REGULATING FACTOR 4 (GRF4) which modulates the balance between growth, N assimilation, and carbon fixation (6–8). In other cases, DELLA proteins increase the transcriptional activity of bound TFs by recruiting transcriptional activation complexes via N-terminal transactivation domains (9–11).

The activity of DELLA proteins is regulated by gibberellins (GAs), a class of phytohormones that promote growth. Although 136 GA forms have been described, only a subset—such as GA_1_ and GA_4_—are biologically active (12). The cellular levels of bioactive GAs are tightly regulated in response to developmental signals and environmental cues through the coordinated activity of different classes of GA biosynthetic and catabolic enzymes (13).

Bioactive GA binds to the receptor protein GIBBERELLIN INSENSITIVE DWARF 1 (GID1), causing a conformational change that enhances its binding affinity for DELLA proteins via conserved N-terminal domains. The resulting GA–GID1–DELLA complex is recognized by the SKP1-CULLIN-F-box (SCF)^GID2^ ubiquitin E3 ligase complex, targeting DELLA proteins for polyubiquitination and rapid 26S proteasome-mediated degradation (14). The GA-mediated degradation of DELLA proteins results in transcriptional changes that promote GA-responsive growth (3).

Mutations in DELLA genes have been exploited in agriculture to improve crop performance. Semi-dwarfing alleles of the B and D homoeologues of *RHT1*, which encode DELLA proteins in wheat (*Triticum aestivum* L.), were selected during the Green Revolution for their positive effects on lodging resistance and assimilate partitioning that results in higher yields in some environments (15). The *Rht-B1b* and *Rht-D1b* alleles carry point mutations that introduce premature stop codons in the N-terminal DELLA domain (4, 16). Translational reinitiation at a downstream AUG codon produces an N-terminally truncated RHT1 protein (ΔRHT-D1) that the GA–GID1 complex cannot bind. This protein is resistant to GA-mediated degradation and confers constitutive partial growth repression in stem tissues (17). In barley (*Hordeum vulgare*), mutations that disrupt the N-terminal DELLA domain of SLENDER1 (SLN1), the orthologous DELLA protein, confer a dominant gain-of-function semi-dwarf phenotype (18). This demonstrates the functional conservation of DELLA genes in temperate cereals.

However, in addition to reduced stem elongation, dominant, gain-of-function *Rht1* and *Sln1* alleles also confer negative pleiotropic effects, such as reduced early vigor and poor nitrogen use efficiency (NUE) that restrict their utility in some environments (19, 20). There is evidence that DELLA proteins regulate growth and development responses such as stem elongation, meristem size, and N-uptake through independent mechanisms controlled by different interacting TFs (8, 21). Therefore, a detailed understanding of the downstream pathways by which DELLA proteins drive different growth responses can help disentangle the effects of DELLA activity to enable more targeted strategies for cereal crop improvement.

Members of the INDETERMINATE DOMAIN (IDD) subclade of Cys2His2 (C2H2) zinc-finger TFs interact with DELLA proteins across monocot and dicot plant species to regulate diverse growth responses (2, 22). IDD proteins contain a conserved N-terminal ID domain consisting of four zinc-finger motifs that mediate DNA binding, and a C-terminal PAM domain required for DELLA interaction (23–26). In *Arabidopsis*, the homologous IDD proteins ENHYDROUS (ENY/AtIDD1) and GAI-ASSOCIATED FACTOR1 (GAF1/AtIDD2) are regulated, in part, through their interactions with DELLA proteins, and act partially redundantly to regulate vegetative growth (27, 28).

In the current study, we demonstrate that the orthologous IDD transcription factors INDETERMINATE DOMAIN 5 (TaIDD5) in wheat and SEMI-DWARF 3 (HvSDW3) in barley physically interact with DELLA proteins and act as positive downstream regulators of GA-dependent stem and leaf expansion. In field trials, although the loss-of-function *idd5* allele confers a GA-insensitive semi-dwarf stature and increased grain number per spike, these lines are lower yielding due to reduced spike number.

## Results

### *HvSDW3* Encodes an IDD Transcription Factor.

**Fig. 1.**
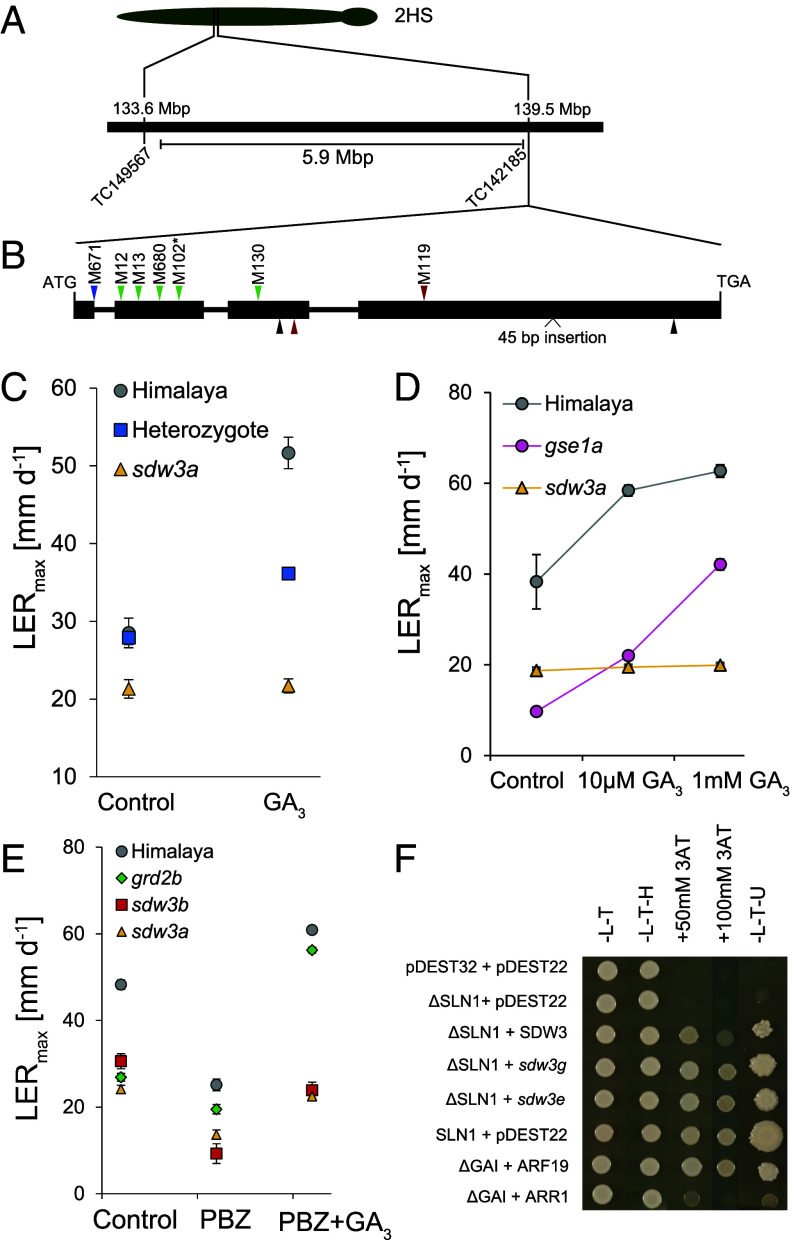
*HvSDW3* encodes an IDD transcription factor. (*A*) *HvSDW3* gene structure showing previous genetic mapping delimiting a 5.9 Mbp interval in the Morex v3 barley genome assembly by placing flanking markers from ref. 29. (*B*) Gene structure of *HvSDW3* showing the position of induced polymorphisms in Himalaya (above the gene model) and the natural mutations in M287 (below the gene model). Different colored arrows indicate the mutation’s functional effect (green = nonsynonymous amino acid substitution, black = synonymous amino acid substitution, blue = splice site mutation, red = premature stop codon). *The mutation in M102 was also found in M107, M127, and M651 (Table 1). (*C*) Maximal rate of first leaf elongation in a population segregating for the *sdw3a* allele in response to 10 μM GA_3_ application. (*N* = 3 to 27 in different genotypic classes). (*D*) Maximal rate of first leaf elongation of *sdw3a* and *gse1a* mutants in response to low (10 μM) and high (1 mM) concentrations of exogenous GA_3_. (*E*) Maximal rates of first leaf elongation of barley mutants in response to treatment with PBZ (10 μM) or PBZ plus GA_3_ (both at 10 μM). Values are means ± SEM *N* = 8. (*F*) Yeast-2 hybrid assays to test the interaction between HvSLN1 and HvSDW3 proteins. GAI + ARF19 and GAI + ARR1 interactions were included as strong and weak positive controls.

We previously mapped *HvSDW3* to a 5.9 Mbp region of chromosome arm 2HS in the barley genome that contains four candidate genes [Fig. 1*A*, (29)]. To determine which of these genes encodes *SDW3*, we sequenced the transcriptomes of wild-type Himalaya, M287 (a Himalaya BC_3_ line carrying the *sdw3* allele), and seven induced mutants derived from Himalaya that exhibit a GA-insensitive semi-dwarf growth habit and which were previously shown to be allelic to *sdw3* by complementation studies (29). Only one gene in the genome (*HORVU.MOREX.r3.2HG0131300*) carried SNPs in all eight sequenced mutants (Fig. 1*B* and *SI Appendix*, Fig. S1). The candidate gene was located at 139,466,760 bp on chromosome arm 2HS of the Morex v3 barley genome assembly, just upstream of the outer proximal flanking marker of the previously identified physical interval [TC142185 139,469,633 bp, *SI Appendix*, Table S1, (29)].

We confirmed the polymorphisms in *HORVU.MOREX.r3.2HG0131300* in the eight mutants by Sanger sequencing and identified four additional induced semi-dwarf mutant lines that also carried mutations in *HORVU.MOREX.r3.2HG0131300*. In total, we identified seven independent *HORVU.MOREX.r3.2HG0131300* alleles that are predicted to disrupt protein function (Table 1). In line M287, *HORVU.MOREX.r3.2HG0131300* carries four polymorphisms in the coding region, including a SNP that introduces a premature stop codon (Table 1).

**Table 1.** Induced and natural allelic variation induced in *HvSDW3*

Line	Allele	Nucleotide change	Effect
M671, M674	*sdw3a*	G91A	Intron 1 splice donor site
M12	*sdw3b*	C214T	S40L (0.00)
M13	*sdw3c*	G277A	R61K (0.01)
M680	*sdw3d*	C409T	P105L (0.00)
M102, M107, M127, M651	*sdw3e*	G424A	G110E (0.00)
M130	*sdw3f*	G843T	E212*
M5	*sdw3g*	C1642T	L404F (0.00)
M287	*sdw3(Hv287)*	A909C	E233D (0.00)
		C1027T	Q273*
		45 bp insertion at 2,316 bp	15 amino acid insertion
		G2914T	Synonymous

Nucleotide and amino acid positions are based on the *SDW3* sequence in Himalaya relative to the translation start codon. Sorting Intolerant From Tolerant (SIFT) scores for amino acid substitutions are shown in parentheses, where values less than 0.05 indicate that the mutation is predicted to have a deleterious effect on protein function. * indicates the introduction of a premature stop codon. Full genomic DNA sequences of all alleles are provided in Dataset S1.

In an M671 x Himalaya F_2_ population segregating for the *sdw3a* allele, seedlings carrying the wild-type *SDW3* allele exhibited a significant increase in leaf extension in response to GA_3_ treatment (*P* < 0.001), whereas those carrying the *sdw3a* allele did not respond (*P* = 0.812, *SI Appendix*, Table S2 and Fig. 1*C*). Heterozygous lines exhibited an intermediate growth response, demonstrating that mutations in *HORVU.MOREX.r3.2HG0131300* are associated with restrictions in cell elongation. A separate assay confirmed that barley seedlings carrying the *sdw3a* allele were insensitive to both low (10 μM) and high (1 mM) concentrations of exogenous GA_3_, in contrast to wild-type seedlings and the GA receptor mutant *gse1a* (Fig. 1*D* and *SI Appendix*, Table S3).

We next tested whether the lack of GA-responsive growth in *sdw3* mutants is due to GA insensitivity or to saturation of the GA response. Treatment with the GA biosynthesis inhibitor paclobutrazol (PBZ) reduced the rate of first leaf elongation of wild-type Himalaya, the GA biosynthesis dwarf mutant *grd2b*, and of the *sdw3a* and *sdw3b* mutants (Fig. 1*E*). Although subsequent GA_3_ application of PBZ-treated seedlings was sufficient to partially restore the rate of first leaf elongation to near control levels in *sdw3a* and *sdw3b* mutants, the effect was far less than in wild-type Himalaya and the *grd2b* mutant, which both exhibited a much greater increase in growth response to exogenous GA_3_ (Fig. 1*E* and *SI Appendix*, Table S4). These results indicate that *sdw3* mutants are not strictly insensitive to GA_3_; rather, under normal growth conditions, one or more of the steps in the GA signaling pathway limit the response.

*HORVU.MOREX.r3.2HG0131300* encodes a C2H2-Zinc finger transcription factor in the IDD subfamily that is most similar to the Arabidopsis proteins ENY and GAF1 (*SI Appendix*, Fig. S2). Both ENY and GAF1 interact with the DELLA protein GAI (24, 27), so we tested whether HORVU.MOREX.r3.2HG0131300 interacts with the barley DELLA protein SLN1. Full-length SLN1 protein exhibits autoactivation, so we tested the interaction using an N-terminally truncated SLN1 protein (∆SLN1) (Fig. 1*F*). The full-length HORVU.MOREX.r3.2HG0131300 protein interacted strongly with ∆SLN1 (Fig. 1*F*). The interaction was not affected by substitutions of conserved amino acids in a region downstream of the PAM domain (L404F in *sdw3g*) or within the ID domain (G110E in *sdw3e*), demonstrating that the reduction in height of these lines is not due to disruption of the SDW3–DELLA interaction (Fig. 1*F* and *SI Appendix*, Fig. S3).

Taken together, our identification of eight independent alleles in *HORVU.MOREX.r3.2HG0131300* that confer reduced growth responses and the role of homologous IDD genes in GA-mediated growth responses (27, 28) led us to conclude that *HORVU.MOREX.r3.2HG0131300* is the causative gene for the *SDW3* locus in barley. The gene will hereafter be referred to as *HvSDW3*.

### IDD5 Interacts with DELLA Proteins in Wheat.

To identify GA signaling components in wheat, we screened an aleurone cDNA library using an ∆RHT-D1 bait protein and identified three independent clones that encode partial sequences matching the A, B, and D homoeologues of *TaIDD5*, the wheat orthologue of *HvSDW3* (*SI Appendix*, Fig. S2). All three encoded proteins included the C-terminal PAM domain and EAR motif but lacked the N-terminal ID domain. The full-length gene sequences encode IDD proteins that share >88% amino acid identity with HvSDW3 (*SI Appendix*, Fig. S4).

**Fig. 2.**
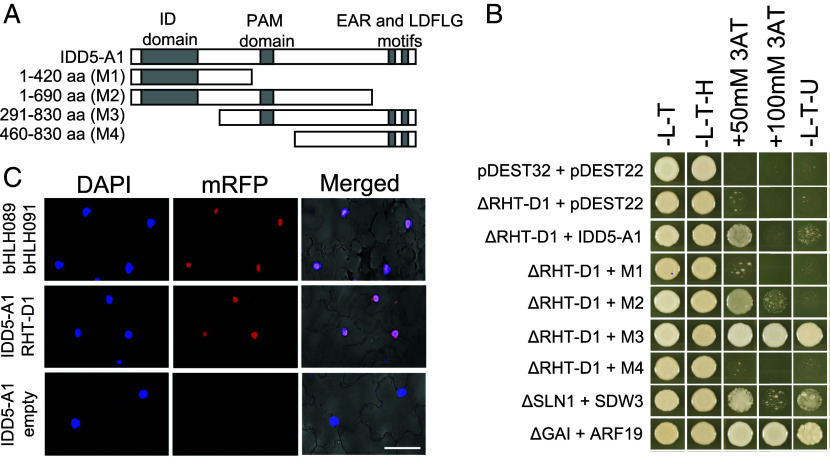
RHT1 interacts with IDD5 in wheat. (*A*) IDD5 proteins tested in Y2H assays. (*B*) Yeast-2 hybrid assays to test the interaction between RHT-D1 and IDD5-A1. GAI + ARF19 and GAI + ARR1 interactions were included as strong and weak positive controls. (*C*) In vivo validation of RHT-D1 – IDD5-A1 interaction using bimolecular fluorescence complementation (BiFC). bHLH089 + bHLH091 coinfiltration serves as a positive control, and TaIDD5-A1 + empty as a negative control. (Scale bar, 50 µM.)

We assembled a series of truncated IDD5-A1 proteins to test the requirement of different domains for the interaction with RHT1 (Fig. 2*A*). We confirmed that full-length IDD5-A1 interacted strongly with ∆RHT-D1 by yeast two-hybrid (Y2H) (Fig. 2*B*) and validated this interaction in vivo using bimolecular fluorescence complementation (BiFC) and DAPI staining, which demonstrated that the interaction occurs in the nucleus (Fig. 2*C*). We assembled a truncated IDD5-A1 protein matching the three cDNA clones identified in the original Y2H screen that lack the INDETERMINATE (ID) domain and found that they exhibit stronger interaction with ∆RHT-D1 than the full-length IDD5-A1 protein (Fig. 2*B*). This indicates that the ID domain is not necessary for the interaction between IDD5 and RHT1, which is consistent with the strong interaction between SLN1 and *sdw3e* (G110E amino acid substitution in the ID domain) (Fig. 1*D*). IDD5 proteins lacking the PAM domain did not interact with RHT1, indicating this domain is required for the interaction (Fig. 2*B*). The interaction between RHT1 and IDD5 proteins lacking the EAR and LDFLG motifs was weaker than with the full-length IDD5 protein, suggesting that these motifs strengthen the interaction but are not necessary (Fig. 2*B*).

Taken together, these results demonstrate that *SDW3* and *IDD5* encode orthologous IDD transcription factors that interact with DELLA proteins in barley and wheat.

### Wheat *idd5* Mutants Exhibit Reduced GA-Insensitive Leaf Elongation.

To characterize *IDD5*, we combined three lines carrying ethyl methanesulfonate (EMS)-induced mutations in *IDD5-A1*, *IDD5-B1,* and *IDD5-D1* to generate an *idd5* mutant carrying loss-of-function mutations in all three homoeologues (Table 2 and *SI Appendix*, Fig. S5*A*). In *IDD5-A1* and *IDD5-D1*, we selected lines carrying mutations that introduce premature stop codons slightly downstream of the PAM domain, which are highly likely to encode nonfunctional proteins (*SI Appendix*, Fig. S5*A*). In *IDD5-B1,* we selected a line carrying a mutation in the intron 1 splice donor site that corresponds to the same mutation in *sdw3a* (Fig. 1*B* and *SI Appendix*, Fig. S1). The mutation in *IDD5-B1* results in a high rate of aberrant splicing, producing transcripts that retain intron 1 and introduce a premature stop codon (*SI Appendix*, Fig. S5*B*). We combined the three mutations and backcrossed three times to wild-type Cadenza to produce a BC_3_F_3_ population segregating for each mutation. We selected lines homozygous for all three wild-type *IDD5* alleles (*IDD5*-null segregant (*IDD5*-NS) and homozygous for all three induced *idd5* mutations (*idd5*) for phenotypic characterization.

**Table 2.** Selected EMS-induced mutations in *TaIDD5* and *RHT1* used in this study

Gene	Gene ID	Position	Effect	TILLING line
*TaIDD5-A1*	*TraesCS2A02G188400*	C1909T	Q492*	CAD4-1185
*TaIDD5-B1*	*TraesCS2B02G218900*	G92A	Intron 1 splice donor site	CAD4-1415
*TaIDD5-D1*	*TraesCS2D02G199300*	C2046T	Q537*	CAD4-0828
*TaRHT-A1*	*TraesCS4A02G271000*	G1845A	W615*	CAD4-350
*TaRHT-B1*	*TraesCS4B02G043100*	G1848A	W616*	CAD4-1509
*TaRHT-D1*	*TraesCS4D02G040400*	G1677A	W559*	CAD4-0587

Position in DNA relative to the translation start codon in Chinese Spring sequences. *indicates the introduction of a premature stop codon.

**Fig. 3.**
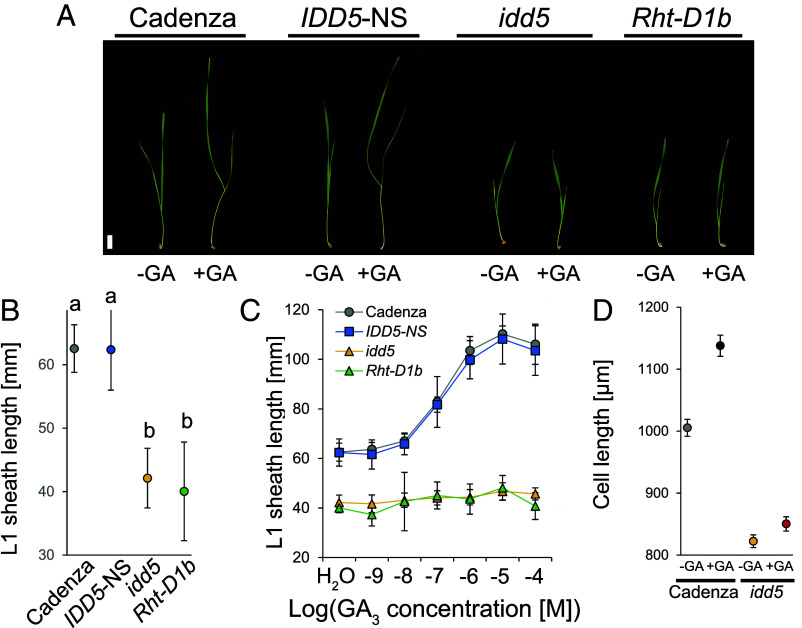
Wheat *idd5* mutants exhibit reduced cell elongation and are GA-insensitive. (*A*) The phenotype of 10-d-old seedlings of Cadenza, *Rht-D1b*, *IDD5*-NS, and *idd5* mutants. Seedlings were either untreated (−) or sprayed with 100 µM GA_3_ every 2 d. (*B*) L1 sheath length in 10-d-old seedlings. Data were analyzed using one-way general ANOVA followed by Tukey’s HSD test. Different letters indicate significant differences between genotypes at the 0.05 confidence level. *N* = 24. (*C*) GA dose–response curves of L1 sheath length of seedlings 14 d after germination. Data were analyzed by one-way ANOVA *N* = 24. (*D*) L1 sheath abaxial epidermis cell lengths of 7-d-old Cadenza and *idd5* seedlings without (−GA) and after 100 µM GA_3_ treatment (+GA). Data were analyzed using unbalanced ANOVA followed by Fisher’s unprotected LSD test. All values are means ± SEM, *N* = 8.

Ten-day-old *idd5* seedlings were shorter than *IDD5*-NS and comparable in height to seedlings carrying the *Rht-D1b* allele (Fig. 3*A*). The first leaf (L1) sheath was significantly shorter in *idd5* seedlings compared to *IDD5*-NS (32.4% reduction, *P* < 0.05), similar to the 36.0% reduction in *Rht-D1b* compared to wild-type Cadenza (Fig. 3*B* and *SI Appendix*, Table S5). Whereas treatment with 100 µM GA_3_ increased the height and L1 sheath length of both Cadenza and *IDD5*-NS seedlings, there was no effect in either *idd5* or *Rht-D1b* genotypes (Fig. 3 *A* and *C*). The L1 sheath elongation responses in both Cadenza and *IDD5*-NS reached saturation in the range of 1 µM to 10 µM GA_3_ (Fig. 3*C* and *SI Appendix*, Table S6). By contrast, neither *idd5* nor *Rht-D1b* mutants responded to any concentration of GA_3_ (Fig. 3*C*). These results were consistent in L1 blade elongation assays (*SI Appendix*, Fig. S6 and Table S7). L1 sheath abaxial cell lengths were significantly different between Cadenza and *idd5* genotypes (*P* < 0.0001), and two-way ANOVA showed a significant interaction between genotype and GA treatment (*P* = 0.18) (Fig. 3*D* and *SI Appendix*, Table S8). The effects on L1 leaf sheath length in this experiment were consistent (*SI Appendix*, Fig. S7). Taken together, these results demonstrate that *idd5* mutants exhibit reduced cell elongation in leaf tissues and are insensitive to GA.

### IDD5 Regulates GA Homeostasis in the Elongating Leaf Sheath.

**Fig. 4.**
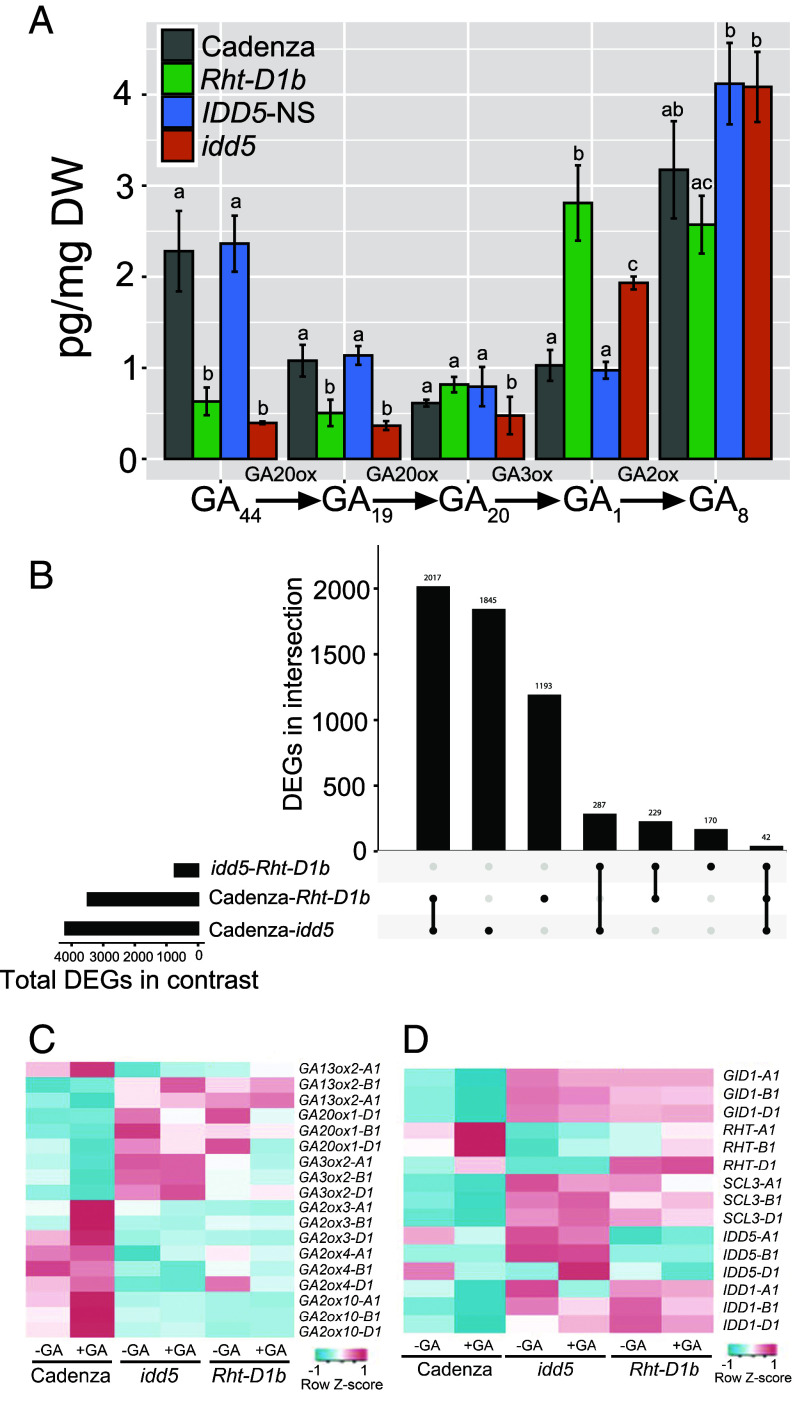
*IDD5* regulates GA levels in the wheat leaf sheath. (*A*) The levels of selected GAs in the leaf sheaths of 7-d-old seedlings of Cadenza, *IDD5*-NS, *idd5,* and *Rht-D1b*. Data were analyzed using one-way ANOVA and Tukey’s HSD test. Different letters indicate significant differences between genotypes (*P* < 0.05). (*B*) UpSet plot showing the number of DEGs in selected pairwise comparisons (*P_adj_* < 0.01) and the subset of those genes that are common to different contrasts. Relative expression levels of selected GA biosynthesis (*C*) and GA signaling (*D*) genes in different genotypes.

We quantified GA levels in elongating wheat leaf sheath and found that all genotypes showed higher 13-hydroxy GA levels than non-13-hydroxy GAs (*SI Appendix*, Table S9) (30), consistent with previous findings that the early 13-hydroxylation pathway predominates in wheat vegetative tissues (31). *Rht-D1b* and *idd5* mutants showed similar GA profiles, with both accumulating significantly higher levels of bioactive GA_1_ (2.7-fold and 2.0-fold, respectively, *P* < 0.001) and lower levels of the GA20OX substrates GA_44_ and GA_19_ than their wild-type segregants (Fig. 4*A*). These GA profiles are consistent with increased GA 20-oxidase activity in both *idd5* and *Rht-D1b* lines. By contrast, only the *idd5* mutant exhibited significantly lower levels of GA_20_, the GA 3-oxidase substrate, consistent with increased GA 3-oxidase activity in this line (Fig. 4*A*).

We sequenced the transcriptomes of Cadenza, *Rht-D1b,* and *idd5* seedlings treated with water or GA_3_ (*SI Appendix*, Fig. S8) (32). In wild-type Cadenza, 364 differentially expressed genes (DEGs) were detected following GA application (Dataset S2, tab 1). By contrast, the GA-insensitive *idd5* and *Rht-D1b* mutants showed only nine and one DEG, respectively (Dataset S2, tabs 2 and 3). Comparing *idd5* and *Rht-D1b* lines revealed 728 DEGs in water-treated seedlings (Fig. 4*B*) and 1,302 DEGs in GA-treated seedlings (*SI Appendix*, Fig. S9). There were approximately five times as many DEGs between Cadenza and either mutant (Fig. 4 *B* and *C*), with significant overlap between transcriptomes; 33.6% of genes differentially expressed between Cadenza and *idd5* are also differentially expressed between Cadenza and *Rht-D1b* (Fig. 4*B*). Taken together, these results indicate that *idd5* and *Rht-D1b* share similar GA profiles and transcriptomes, suggesting both mutated genes act in the same genetic pathway.

In Cadenza, GA treatment reduced the expression of the GA biosynthetic genes *GA3OX2* and *GA20OX1* and increased the expression of the GA catabolic genes *GA2OX3, GA2OX4,* and *GA2OX10*, consistent with their feedback regulation in maintaining GA homeostasis (Fig. 4*C*). By contrast, *GA3OX2* and *GA20OX1* transcript levels were higher in both *idd5* and *Rht-D1b* than in Cadenza and were unaffected by GA treatment (Fig. 4*C*). These results are consistent with elevated GA_1_ levels and the reduced levels of the GA20OX1 substrates GA_44_ and GA_19_ (Fig. 4*A*). Similarly, *GA2OX3*, *GA2OX4,* and *GA2OX10* transcript levels were reduced in *idd5* and *Rht-D1b* mutants and did not increase after GA treatment, suggesting a disruption in GA homeostasis.

In the GA signaling pathway, both the GA receptor *GID1* and *SCARECROW-LIKE 3* (*SCL3*) were upregulated in *idd5* and *Rht-D1b* compared to Cadenza (Fig. 4*D*), while *Rht1* genes were downregulated in *idd5* (Fig. 4*D*).

### TaIDD5 and HvSDW3 Are Epistatic to DELLA.

**Fig. 5.**
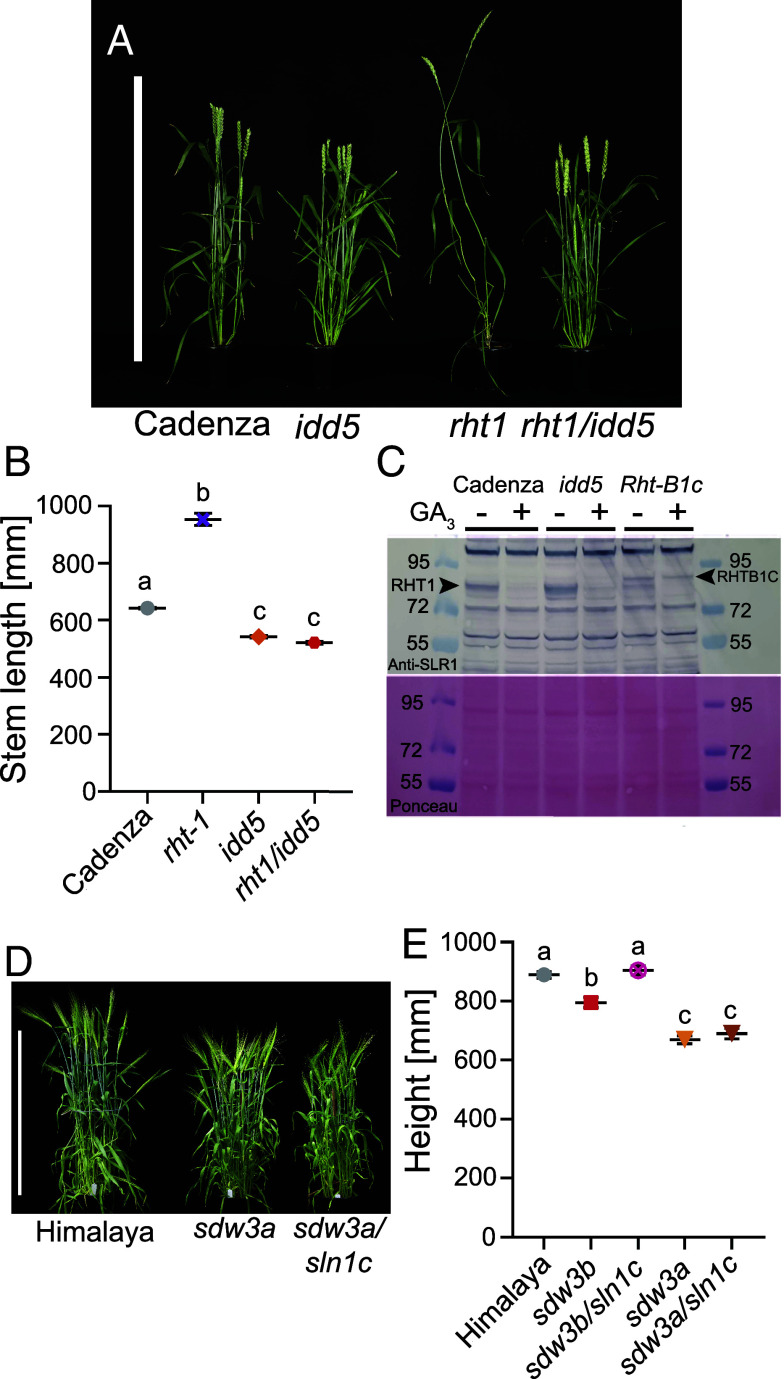
*IDD5* and *SDW3* act downstream of DELLA genes. (*A*) Whole plant phenotypes of *rht1, idd5,* and *rht1/idd5* mutants at maturity. (*B*) Mean stem length (mm) ± SEM at maturity (*N* = 18). (*C*) Detection of RHT1 proteins by western blot in Cadenza, *idd5,* and *Rht-B1c* immature spike protein extracts. Protein extracts were untreated or treated with GA_3_. RHT1 and RHT-B1C proteins are indicated. (*D*) Whole plant phenotypes of *sdw3a* and *sdw3a/sln1c* mutant barley plants at maturity. (*E*) Total plant height at maturity. Data were analyzed with one-way ANOVA and Tukey’s post hoc HSD test. Different letters indicate significant differences between genotypes at the 0.05 confidence level. *N* = 11 to 12. (Bar, 90 cm.)

To test the epistatic interactions between *IDD5* and *RHT1*, we developed an *rht1* loss-of-function mutant by combining three lines carrying induced mutations introducing premature stop codons in the C-terminal SAW domain of each *RHT1* homoeologue (Table 2). The resulting *rht1* mutant is sterile and exhibits a low-tillering, spindly growth habit resembling that of loss-of-function DELLA mutants in other species (Fig. 5 *A* and *B*). We crossed *rht1* with *idd5* to generate a sextuple *idd5*/*rht1* mutant. In the *idd5* background, the spindly, low-tillering phenotype of *rht1* was rescued, and the height of the *idd5*/*rht1* mutant was not significantly different from *idd5* (*P* > 0.05, Fig. 5 *A* and *B* and *SI Appendix*, Table S10). Despite rescuing the height phenotype, both the *rht1* and *idd5*/*rht1* mutants remained infertile.

In wild-type Cadenza, native RHT1 proteins in immature spike extracts are degraded in response to GA application (Fig. 5*C*). By contrast, in the GA-insensitive *Rht-B1c* mutant, the RHT-B1C protein—which contains a 90 amino acid insertion in the N-terminal DELLA domain (16)—is not degraded after GA application (Fig. 5*C*). In the *idd5* mutant, DELLA proteins are still degraded in response to GA, demonstrating that reduced stem elongation in this line is independent of RHT1 degradation.

We next tested epistatic interactions between SLN1 and SDW3 by introducing the loss-of-function *sln1c* allele into selected *sdw3* backgrounds. In a Himalaya background, the *sln1c* allele causes excessive elongation growth of all aerial parts and confers male sterility (18). When combined with the *sdw3a* allele, elongation growth was markedly reduced (Fig. 5*D* and *SI Appendix*, Table S11), and male fertility was restored, as indicated by a full grain set.

Although plant height was not significantly different between *sdw3a* and *sdw3a/sln1c* mutants, the *sdw3b/sln1c* mutant was significantly taller than *sdw3b* (Fig. 5*E* and *SI Appendix*, Table S11). This suggests that *sln1c* can partially rescue *sdw3b* to near wild-type height, although to a much smaller degree than in a wild-type background (18). Consistent with this, we observed slight elongation of the subcrown internode in *sdw3b/sln1c*—a characteristic response of *sln1c* in a wild-type background—but not in the *sdw3a* background (*SI Appendix*, Fig. S10). These observations likely reflect that *sdw3b* (S40L) retains a low degree of SDW3 activity, compared to the more severe splice-site mutation in *sdw3a* (Table 1), consistent with a smaller reduction in height (see below).

### *idd5* and *sdw3* Mutants Exhibit a Semi-Dwarf Phenotype.

**Fig. 6.**
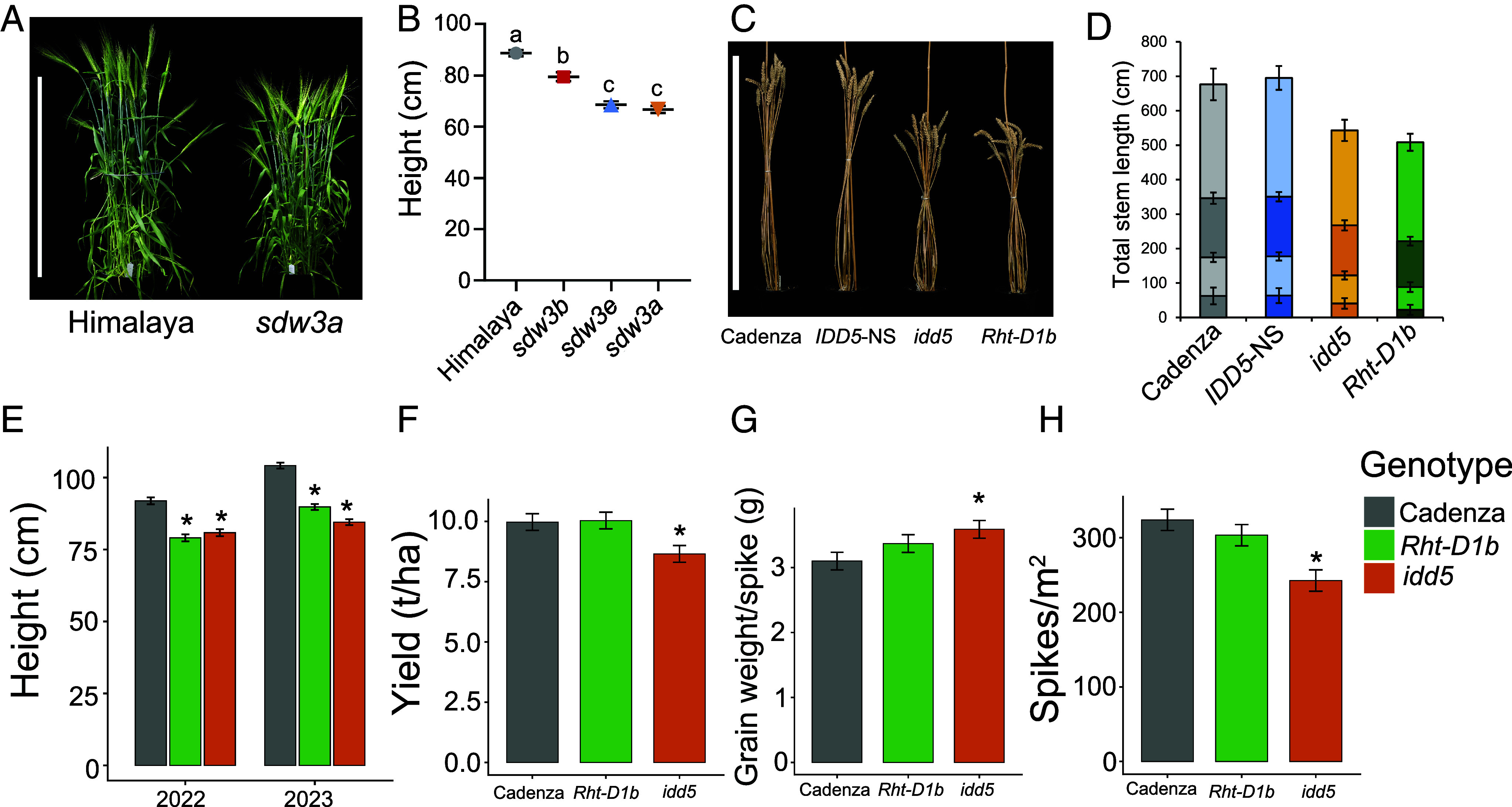
The phenotype of the mature barley *sdw3* mutants and wheat *idd5* mutants. (*A*) Mature phenotype of wild-type Himalaya and *sdw3a* (M671). (B) Mean height ± SEM of wild-type, *sdw3a, sdw3b,* and *sdw3e* mutants (*N* = 11 to 12). Different letters indicate statistically significant differences determined by one-way ANOVA followed by Tukey’s post hoc test (*P* < 0.05). (*C*) Mature phenotype of wheat genotypes grown in the glasshouse. (*D*) Mean length of representative tilers from each genotype with internodes indicated. *N* = 24. The wheat phenotypic data collected in glasshouse experiments were analyzed using one-way ANOVA and mixed linear models followed by Tukey’s post hoc LSD test (*P* < 0.05). Plotted values are means ± SEM. Bars in panels *A* and *C* are 80 cm. (*E*–*H*) Phenotypic data from Cadenza, *Rht-D1b,* and *idd5* lines from 2022 and 2023 field experiments. Height exhibited a significant trial × genotype interaction, so data from each experiment are presented separately. Yield, grain weight, and spike number are presented as the joint values across both experiments. Data were analyzed with linear mixed models. Values are means ± SEM, *N* = 4. * = significant difference with Cadenza (*P* < 0.05).

In glasshouse conditions, the barley *sdw3a* allele reduced plant height by 24.3% compared to wild-type Himalaya (Fig. 6 *A* and *B* and *SI Appendix*, Table S12). The *sdw3e* allele conferred a similar reduction in plant height (22.6%), whereas the *sdw3b* allele had a milder effect (10.3% reduction, Fig. 6*B* and *SI Appendix*, Table S12).

Wheat plants carrying loss-of-function mutations in one or two *IDD5* homoeologues exhibit only mild reductions in height in glasshouse conditions (*SI Appendix*, Fig. S11 and Table S13). By contrast, the *idd5* triple mutant reduced total stem length by 21.5% compared to *IDD5*-NS (*P* < 0.001) due to decreased elongation of all internodes (Fig. 6 *C* and *D* and *SI Appendix*, Table S14). The effect was milder than that of the *Rht-D1b* allele, which conferred a 25.9% reduction in stem length compared to Cadenza (*P* < 0.001). All internodes in *idd5* except the peduncle were significantly longer than in *Rht-D1b* (Fig. 6*D* and *SI Appendix*, Table S14). Coleoptile length was significantly reduced in both *idd5* and *Rht-D1b* mutants compared to Cadenza, but neither seedling root length nor germination rate was affected (*SI Appendix*, Table S15).

We evaluated Cadenza, *Rht-D1b,* and *idd5* genotypes in field plots over two consecutive seasons. Both *Rht-D1b* and *idd5* were significantly shorter than Cadenza in each year (*P* < 0.05, Fig. 6*E* and *SI Appendix*, Table S16). Plant height was reduced by 12.9 to 14.4 cm in *Rht-D1b* and by 11.1 to 19.6 cm in *idd5*, caused in both genotypes by significant reductions in the lengths of all internodes (*SI Appendix*, Fig. S12). The spike was significantly longer in *idd5* mutants than Cadenza (*P* < 0.05) but unaffected in *Rht-D1b* (*SI Appendix*, Fig. S12).

Despite similar height reduction, grain yield was 13.2% lower in *idd5* mutants than in Cadenza (*P* < 0.05), whereas *Rht-D1b* exhibited a slight increase (Fig. 6*F* and *SI Appendix*, Table S16). To understand the basis of this yield reduction, we phenotyped individual yield components. Spikelet number was significantly higher in *idd5* than in Cadenza, and both *Rht-D1b* and *idd5* produced more grains per spike (Fig. 6*G* and *SI Appendix*, Table S16). In *Rht-D1b*, all sections of the spike showed improved fertility, whereas in *idd5*, the effect was greatest in basal spikelets (*SI Appendix*, Fig. S13). Grain weight per spike was significantly increased in both *idd5* and *Rht-D1b*, demonstrating that both alleles confer improved yield per spike (Fig. 6*G*). However, spike number was significantly reduced in *idd5* mutants, but not in *Rht-D1b* (Fig. 6*H*). Overall, although *idd5* confers height reduction and greater yield per spike similar to *Rht-D1b*, these gains are offset by a significant decrease in spike number, resulting in lower overall yields.

## Discussion

### Dissecting Growth Pathways in Cereals.

Functional diversification among DELLA-interacting TFs and their downstream targets likely contributes to the diversity in tissue-specific GA responses (2). Despite the importance of Green Revolution DELLA alleles in modern agriculture, the key interacting TFs in crop species remain poorly understood (33). In *Arabidopsis*, at least seven IDD proteins bind DELLA (22) and the AtIDD4–DELLA interaction is conserved in lycophyte and bryophyte lineages (2), indicating that DELLA-mediated regulation of IDD proteins is an ancestral mechanism. Several IDD proteins regulate growth, including the homologues BROAD LEAF1 in barley and AtIDD14/15/16 in *Arabidopsis* that repress longitudinal cell division in leaves (34). In *Arabidopsis*, GAF1 and ENY (homologues of IDD5 and SDW3) act partially redundantly to promote vegetative growth (24, 27). Consistent with this, both the wheat and barley genomes contain paralogues of *IDD5* and *SDW3* that encode proteins with a high level of sequence identity and included an ID domain and EAR motif (*SI Appendix*, Figs. S2 and S3), suggesting potential functional redundancy in cereals. Generating loss-of-function alleles in these paralogues in wheat and barley will help characterize their function and test this hypothesis.

Our finding that *idd5* and *sdw3* loss-of-function mutations confer semi-dwarfism indicates that they are key components of elongation growth pathways in cereals and provides an entry point to better understand these processes. To dissect these regulatory networks, it will be important to identify the direct genomic loci targeted by IDD5 and SDW3 and to determine how their transcriptional activity is affected by DELLA proteins. One model is that DELLA proteins sequester or inhibit IDD5/SDW3, similar to their repression of the positive growth regulator PIF4 (6, 7). GA-induced DELLA degradation would then release IDD5/SDW3 to promote growth. Alternatively, DELLAs may enhance IDD transcriptional activity by recruiting coactivator complexes (9, 11), potentially increasing the expression of growth-inhibitory genes.

Direct IDD5 targets might include GA biosynthesis and signaling genes. In *Arabidopsis*, the GAF1–GAI complex binds the promoters of *AtGA20OX2*, *AtGA3OX1,* and *AtGID1b* activating their expression (25, 27). In peach [*Prunus persica* L. (Batsch)], an IDD1–DELLA complex activates *GA20OX1* expression (35), suggesting a conserved role for IDD–DELLA complexes in GA homeostasis. In wheat, *GA3OX2* and *GA20OX1* transcript levels were increased in *idd5* mutants and reduced in response to GA treatment, supporting the hypothesis that these genes are direct IDD5 targets modulated by DELLA activity (Fig. 4*C*).

The increased transcript levels of GA feedback-regulated genes and higher bioactive GA levels in the *idd5* mutant are unexpected, given that IDD5 promotes GA-responsive growth (Fig. 4). One explanation is that, in the absence of IDD5, DELLA proteins are no longer sequestered and can act as coactivators of other transcriptional regulators at GA biosynthesis gene loci. Other factors, such as the non-DELLA GRAS protein SCL3 (28), might also regulate transcription of these IDD5 targets. The opposing role of OsIDD2 as a negative regulator of GA-mediated cell elongation in rice (36) suggests that different IDD proteins may act antagonistically by competing at shared promoters to fine-tune expression of GA homeostasis genes.

### Application of *IDD5* and *SDW3* Alleles in Agriculture.

The ability of *idd5* and *sdw3* loss-of-function mutations to fully rescue the excessive elongation of *della*-null mutants demonstrates the central role of IDD5/SDW3 TFs in regulating stem elongation in wheat and barley. Because DELLA proteins in these lines remain responsive to GA-mediated degradation (Fig. 5), we hypothesized that *idd5/sdw3* alleles could confer the benefits of semi-dwarf stature without the negative pleiotropic effects associated with Green Revolution dwarfing alleles. However, when *idd5* lines were tested in the field, increases in spike length, spikelet number, and grain number were offset by a reduction in spike number, resulting in a significant yield penalty (Fig. 6). Grains from *idd5* exhibited a high germination rate in laboratory assays (*SI Appendix*, Table S15), suggesting that altered vegetative development leading to fewer productive tillers is a likely cause of reduced spike number and thus yield.

We also found that *idd5* alleles did not overcome several pleiotropic effects associated with *Rht-D1b*. Both semi-dwarfing alleles conferred shorter coleoptiles, suggesting that *idd5* lines might suffer weak emergence when sown deeply in hot, dry soils. Furthermore, because a uniform N application rate was used for all genotypes, the yield reduction in *idd5* indicates that it exhibits poorer NUE relative to *Rht-D1b* (Fig. 6). Taken together, although only tested in a single genetic background, our results suggest that *idd5* has limited value as an alternative semi-dwarfing allele for wheat breeding.

We did not evaluate barley *sdw3* alleles in field trials in the current study. Analysis of 44 barley pangenome accessions shows that all carry *SDW3* alleles encoding full-length proteins with intact functional domains (*SI Appendix*, Table S17) (37), suggesting that *sdw3* loss-of-function mutations have not been widely exploited in barley breeding. Introducing such alleles using CRISPR/Cas9-based gene editing could, therefore, be a straightforward approach to engineer novel semi-dwarfing traits. Additionally, screening diverse germplasm might uncover additional natural *SDW3* alleles, including partially functional alleles such as *sdw3b*, which confer milder height reductions (Fig. 6*B*). Such variation could allow breeders to tailor height reductions to specific environments or agronomic requirements. It is interesting to note that *sdw3* mutations restore fertility in barley *sln1c* loss-of-function mutants, whereas the equivalent *rht1/idd5* line in wheat remains sterile. This suggests either functional divergence between SDW3 and IDD5 or the involvement of different IDD proteins in reproductive development in each species.

Our study highlights the importance of replicated field testing to support claims of improved crop performance when reporting novel genetic variants (38). Growing lines in large plots under standard UK agronomic practice allowed us to gather robust yield measurements, while our analysis of yield subcomponents clarified how these alleles affect distinct developmental processes. Although *idd5* alleles are likely to be poor candidates to directly replace Green Revolution semi-dwarfing genes, our findings provide avenues for future research into growth pathways in cereals, including downstream targets that may overcome the negative pleiotropic effects of these alleles.

## Materials and Methods

### Plant Materials and Growth Conditions.

All wheat lines were in the Cadenza background. Near-isogenic lines carrying *Rht-D1a* and *Rht-D1b* alleles were described previously (17). EMS-mutagenized M_4_ lines carrying *IDD5* or *RHT1* mutations were identified from an in silico database (39). M_4_ plants of each *IDD5* mutant line were intercrossed and then backcrossed three times to Cadenza. From the resulting BC_3_F_2_ populations, *IDD5*-NS and *idd5* genotypes were selected using assays described in *SI Appendix*, Table S18. To determine the effect of the intron 1 donor splice site mutation in *IDD5-B1*, PCR amplicons spanning exons 1 and 2 of all three *IDD5* homoeologues were amplified using primers described in *SI Appendix*, Table S18. Illumina sequencing libraries were generated from these pooled amplicons and sequenced at the Rothamsted Research sequencing core. Reads were aligned to *IDD5* gene models using Bowtie 2, and the proportion of correctly versus incorrectly spliced reads was calculated from read pileups (*SI Appendix*, Fig. S5*B*).

Similarly, M_4_ plants from each *RHT1* mutant line were backcrossed twice to Cadenza and then crossed to combine the mutations, producing BC_2_F_2_ lines homozygous for knockouts in *RHT-A1* and *RHT-B1* and heterozygous for the *RHT-D1* mutation. These lines were maintained in a heterozygous state in *RHT-D1* to overcome the sterility of the *rht1*-null mutant. BC_2_F_3_ plants homozygous for all three *RHT1* knockouts were used in subsequent experiments. Crosses between *rht1* and *idd5* mutants resulted in the generation of the sextuple *idd5/rht1* line.

Initial phenotyping was performed in the glasshouse at 20 °C using a 16 h/8 h light/dark cycle. All other experiments were conducted in controlled environment growth conditions under a 16 h photoperiod (300 µmol m^−2^s^−1^) with day/night temperatures of 20 °C/16 °C. One-hundred grains of Cadenza, *Rht-D1b,* and *idd5* lines were sterilized with 15% bleach, rinsed, and placed in germination paper. Seeds were incubated at 4 °C overnight, then transferred to a controlled environment of 22 °C maintained in darkness for 10 d, after which germination rate, coleoptile length, and root length from each seedling were measured.

All barley materials were in the Himalaya background. To identify *SDW3* mutants, wild-type Himalaya grains were mutagenized with sodium azide (40) and then sown in the field. Bulk M_2_ grains were harvested and screened in soil in wooden flats that were watered with 1 µM GA_3_ solution. Most seedlings were highly elongated and pale green in response to GA_3_ treatment, but approximately 1 in 1,000 seedlings were dark green and semi-dwarf. These semi-dwarf plants were transplanted to pots and grown to maturity without further GA treatment. Intercrossing of these lines over subsequent generations indicated that they included several different genetic loci. Ten mutants defined by M671 formed a single complementation group (29). Further complementation tests with previously described dwarf barley mutants showed that M671 is allelic to the dwarfing mutation in Hv287, originally designated *GA-insensitive* (*GA-ins*) (41) but now renamed *Sdw3* (42). All *sdw3* alleles were confirmed by Sanger sequencing of three overlapping PCR amplicons (*SI Appendix*, Table S18). In the Himalaya x M671 and *sdw3a* x *sln1c* populations, *sdw3a* was genotyped by Sanger sequencing of the amplicon generated from primers Sdw3_F1/Sdw3_R1 (*SI Appendix*, Table S18), and *sln1c* was genotyped by Sanger sequencing of PCR amplicons as described previously (18). Other barley mutants used in this study included *gse1a* (M488), which carries an amino acid substitution in the HvGID1 GA receptor (43, 44), *grd2b* (M463), which is GA-deficient due to a loss-of-function mutation in *HvGA3OX2* and *sln1c* (18). All barley plants were grown under standard glasshouse conditions at 20 °C with a 16 h/8 h light/dark cycle.

### Field Experiments.

Cadenza, *Rht-D1b,* and *idd5* genotypes were sown in autumn 2021 (11/10/2021) and autumn 2022 (4/10/2022) at the Rothamsted Experimental farm in Southeast England in 9 × 1.8 m plots arranged in a randomized block design at a rate of 450 seeds/m^2^. The field trial plan comprised 128 plots arranged in four replicate blocks of 4 rows and 8 columns, with the complete trial arranged as 4 rows by 32 columns. Lines (32 in total) were allocated to plots according to a resolvable row-column design with blocking as described and additional Latinization across “long rows” (i.e., single rows of 32 plots cutting across the replicate blocks) to allow for potential additional variation due to the direction of farming operations (perpendicular to spatial blocking). Standard farm practice for fertilizer and pesticides was followed, with no plant growth regulators applied. Height measurements were made using a floating polystyrene disc, taking six measurements per plot. Internode lengths were measured for ten individual tillers by measuring between the lower bounds of each node. Spikelet number and grain number per spikelet were counted from ten representative spikes, including values for basal (spikelets 1 to 8), central (spikelets 9 to 17), and apical (spikelets 17 and above) sections of the spike. Grain yield was measured after final harvest at 85% dry matter, calculated using the fresh weight of grain per plot and the grain dry matter content. Dry matter was calculated from the fresh and dry weight of a subsample of approximately 80 g, dried for 16 h at 105 °C, which was also used to calculate thousand-grain weight (TGW). Grain weight per spike was calculated from grain number and TGW, while spike number was derived from plot yield and grain weight per spike values.

Data were analyzed using linear mixed models fitted using restricted maximum likelihood, each with a one-way fixed model (line) and the random model reflecting the design/randomization structure of the trial ((block/row)×long row). While data for all lines grown were included in the analysis results for comparisons between only the three lines of interest are given here (extracted using a nested treatment contrast). Traits measured in both field trials were first analyzed for each trial separately using linear mixed models (LMMs) fitted using restricted maximum likelihood (REML), each with a one-way fixed model (line) and the random model reflecting the design/randomization structure of the trial ((block/row)×long column in each case). Data from both trials were then combined using an LMM meta analysis with a two-way (trial×line) fixed model and allowing a separate random model for each trial.

### Cell Length Measurements.

Two images of abaxial epidermis cells were taken in the uppermost 10 mm of the fully elongated leaf sheath using a JEOL JSM-6360LV scanning electron microscope. Twelve plants per genotype and treatment were analyzed. All visible individual cells in each image were measured with ImageJ 1.48v software (45). The data were compared using unbalanced ANOVA with Tukey’s post hoc HSD test.

### GA Dose–Response Assays.

Wheat seeds were surface sterilized, germinated, and transplanted into vermiculite saturated with water or GA_3_ solutions ranging from 1 nM to 100 µM in 10-fold increments. Plants were treated every 2 d for 10 d, after which seedlings were harvested and the first leaf sheath and blade lengths were measured. Treatments were replicated three times in a blocked design. Barley seeds were germinated in filter paper envelopes soaked with either water or GA_3_ solutions. The maximum leaf elongation rate (LER_max_) of each seedling was determined following the method described previously (44). Genotypes with a normal GA response reach near-saturation of leaf elongation at GA_3_ concentrations of 1 to 10 µM (44). By contrast, the *gse1* mutant is less sensitive to GA and shows continued increases in leaf elongation at very high (mM) concentrations of GA_3_ (43). All measurements were analyzed using one-way ANOVA, accounting for replication and blocking in a nested treatment structure (Block/Unit). The least significant difference (LSD) was set at the 5% level. GenStat (20th edition, 2019, ©VSN International, Hemel Hempstead, UK) was used for all analyses, and residual and mean plots were examined to confirm data normality.

### Protein–Protein Interaction Assays.

A wheat cDNA library was constructed from RNA extracted from mature aleurone tissues. Grains were de-embryonated and incubated for 3 d in 20 mM CaCl_2_, after which the aleurone layers were isolated from half-grains. Total RNA was extracted and used to generate cDNA prey libraries (Life Technologies Corporation). This library was screened using a plasmid containing Δ*RHT-D1* (nucleotides 652 to 1,872, amino acids 218 to 623) described previously (16). To transform the library, 250 μL of library-scale MaV203 competent cells (>1 × 10^6^ transformants, Thermo-fisher Scientific, California, USA) was mixed with 10 μg of bait plasmid, 10 μg of wheat aleurone cDNA prey library and 1.5 mL of PEG/LiAc solution. The mixture was incubated at 30 °C for 30 min, then treated with 88 μL DMSO (Sigma-Aldrich, Darmstadt, Germany) and heat-shocked at 42 °C for 20 min. Pairwise interactions were tested in a Y2H system with plasmids expressing *IDD5-A1*, Δ*SLN1* (nucleotides 640 to 1,857, amino acids 214 to 619), *SDW3*, and truncated forms of *TaIDD5-A1* (M1, M2, M3, and M4). Plasmids were introduced into *MaV203* yeast competent cells by heat shock. 150 µL of competent yeast cells were incubated with 1 µg of each plasmid DNA, 2 µL of 10 mg/mL sheared salmon sperm DNA (Thermo-fisher Scientific, California, USA), and 350 µL of 50% polyethylene glycol (PEG3350) at 30 °C for 30 min, then 42 °C for 5 min, placed on ice for 2 min and centrifuged. Cell pellets were resuspended in 110 µL of sterile distilled water and plated on SD-Leu-Trp selective media, then incubated at 30 °C for 72 h. A small amount of yeast colony originating from a single cell was resuspended in 200 µL of sterile water. A 5 µL aliquot of this suspension was spotted onto SD-Leu-Trp-His medium containing 50 and 100 mM of 3-AT and onto SD-Leu-Trp-Ura medium. Three biological replicates were performed for each strain, and plates were incubated at 30 °C for 72 h to assess relative growth.

For bimolecular fluorescence complementation, destination vectors (AB830561, AB830564, AB830568, AB830572) used in this study were described previously (46). Full-length *RHT-D1* and *IDD5-A1* coding sequences were inserted into these vectors using Gateway cloning. The *IDD5-A1* gene was synthesized by GenScript (Netherlands), and the codons were optimized for expression in *Nicotiana benthamiana*. Cultures of *Agrobacterium tumefaciens* strain GV3101 carrying the fusion constructs were grown overnight in 2YT media containing 50 µg/mL rifampicin, 25 µg/mL gentamicin, and 100 µg/mL spectinomycin. 1 mL of each culture was pelleted (1,505×*g* for 5 min), resuspended in infiltration medium (28 mM D-glucose, 50 mM MES, 2 mM Na_3_PO_4_·12H_2_O, 100 µM acetosyringone) to an OD_600_ of 0.1, and mixed 1:1:1 with a p19 control culture. The resulting suspensions were infiltrated into the abaxial sides of leaves from 6- or 7-wk-old *N. benthamiana* plants. After 3 d, infiltrated leaves were examined using a Zeiss LSM 780 confocal microscope with Leica Application Suite X (LAS X) software.

### Western Blot.

Proteins were extracted from developing wheat spikes by homogenizing the tissue in an extraction buffer containing one cOmplete™ ULTRA Mini EDTA-free Protease Inhibitor Cocktail Tablet (Merck KGaA, Darmstadt, Germany), 2 mL of 5× lysis buffer (50 mM Tris/Cl pH 7.5, 750 mM NaCl, 2.5 mM EDTA) and 100 µL of 100 mM PMSF. After a 20 min incubation, samples were denatured at 70 °C for 10 min, separated by SDS-PAGE, and transferred onto 0.2 µm PVDF membranes. Membranes were first stained with Ponceau S to confirm equal protein loading, then blocked and incubated overnight (16 h at 4 °C) with rabbit anti-SLR1 primary antibody (1:5,000). Following washes, membranes were incubated for 4 h at 4 °C with goat anti-rabbit IgG alkaline phosphatase (1:5,000 dilution, Merck) before developing with the BCIP®/NBT-Purple Liquid Substrate System (Merck) for up to 2 h.

### GA Hormone Extraction and Analysis.

Aerial tissues of 7-d-old seedlings of Cadenza, *IDD5*-NS, *idd5,* and *Rht-D1b* (four biological replicates per genotype) were harvested from the grain to the top of the coleoptile, frozen in liquid N_2_ and freeze-dried. Sample preparation and analysis were performed according to Urbanová et al. (47) with some modifications. Briefly, about 5 mg DW was homogenized in 1 mL of ice-cold 80% aqueous acetonitrile containing 5% formic acid using 2.7-mm ceria stabilized zirconium oxide beads (Next Advance Inc., USA) and an MM 400 vibration mill at a frequency of 27 Hz for 3 min (Retsch GmbH & Co., Germany). The samples were then extracted overnight at 4 °C using a benchtop laboratory rotator Stuart SB3 (Bibby Scientific Ltd., UK) after adding 2 pmol of [^2^H_2_]GA_1_, [^2^H_2_]GA_4_, [^2^H_2_]GA_9_, [^2^H_2_]GA_19_, [^2^H_2_]GA_20_, [^2^H_2_]GA_24_, [^2^H_2_]GA_29_, and [^2^H_2_]GA_44_ (OlChemIm, Czech Republic) as internal standards. The homogenates were centrifuged at 36,670×*g* and 4 °C for 10 min (Hermle Z 35 HK, Hermle Labortechnik GmbH, Germany), and the supernatants were purified using mixed-mode SPE cartridges (Oasis® MAX, 60 mg/3 mL; Waters, Ireland) as described in ref. 47. After SPE purification, the samples were evaporated to dryness in vacuo (CentriVap® Acid-Resistant benchtop concentrator, Labconco Corp., USA), redissolved in the starting mobile phase (MeOH: 10 mM formic acid, 1:9 (v/v)) and analyzed by ultra-high performance liquid chromatography–tandem mass spectrometry (UHPLC–MS/MS) using an Acquity UPLC I-Class Plus system (Waters, USA) coupled to a triple quadrupole mass spectrometer Xevo TQ-XS (Waters, USA). GAs were separated on an Acquity UPLC CSH C18 column, 2.1 × 50 mm, 1.7 µm (Waters, Ireland), eluted with a linear gradient from 1:9 to 6:4 MeOH-10 mM formic acid (v/v) over 15 min at 0.25 mL/min. The MS settings were as follows: capillary voltage 1.5 kV, cone voltage 30 V, source temperature 150 °C, desolvation gas temperature 600 °C, cone gas flow 150 L/h and desolvation gas flow 1,000 L/h. GAs were detected using multiple-reaction monitoring mode of the transition of the ion [M–H]^−^ to the appropriate product ion (for settings of individual transitions see ref. 47). Masslynx 4.2 software (Waters, USA) was used to analyze the data. The quantitation of GA levels was performed based on the standard isotope dilution method (48). Data were statistically processed using one-way ANOVA and Tukey’s post hoc HSD test.

### Transcriptome Analysis.

Leaf sheaths from 7-d-old Cadenza, *idd5,* and *Rht-D1b* plants (four biological replicates each) were collected 8 h after treatment with either water or 100 µM GA_3_. Tissue from the grain to the top of the coleoptile was harvested, flash-frozen in liquid nitrogen and total RNA was extracted using the Monarch® Total RNA Miniprep Kit with on-column DNase treatment (New England Biolabs, Ipswich, MA). RNA quality was assessed using an Agilent RNA 6000 Nano Chip and Agilent 2100 Bioanalyzer (Agilent, Santa Clara, USA).

RNA samples were sequenced using paired-end Illumina sequencing by Novogene Europe (Cambridge, UK). Sequencing reads were trimmed for quality and adapter sequences using Trimmomatic 0.39 (parameters SLIDINGWINDOW:4:20; MINLEN:50) (49), then aligned and quantified using Kallisto against the IWGSC RefSeq v1.2 annotated gene models (47, 50). Transcript per million (TPM) values were calculated for each gene, and pairwise contrasts were made between control and GA_3_ treatments for each genotype, as well as between genotypes for the same treatment using DESeq v2 (51). UpSet plots (52) were used to visualize DEGs, and TPM values of selected genes were displayed with the online tool Heatmapper (http://www.heatmapper.ca/expression/).

Total RNA was extracted from young barley shoots grown in filter paper envelopes and sequenced as 2 × 150 bp paired end Illumina sequencing by Novogene (China). Trimmomatic (49) was used to remove low-quality bases and adapter sequences, and the resulting high-quality paired reads were mapped to Morex v2 barley reference gene models (53) using Tophat2/Bowtie2 tools (bowtie/2.3.4 and tophat/2.1.1; (54) with default parameters. Sorting BAM files and removing duplicate reads was performed as described previously (55). Single nucleotide polymorphism (SNP) calling and candidate gene identification were performed using the MUTRIGO pipeline (https://github.com/TC-He-witt/MuTrigo) with default settings.

### Phylogenetic Analysis.

Putative IDD family members in wheat and barley were identified using BLASTp searches against the IWGSC RefSeq v1.2 and Morex V3 proteome databases through the Ensembl Plants platform (https://plants.ensembl.org/index.html). Rice IDD family members and the IDD were used as search queries. Phylogenetic analysis was conducted in PhMyl (56) with a BLOSUM62 substitution model and 100 bootstrap replicates to generate a rooted tree using a *Marchantia polymorpha* IDD protein encoded by *Mp5g20530* as an outgroup. Protein alignments were produced using MUSCLE (54). SDW3 proteins were identified using BLASTp from the proteomes of 44 barley accessions from the barley pangenome (37).

## Supplementary Material

Appendix 01 (PDF)

Dataset S01 (PDF)

Dataset S02 (XLSX)

## Data Availability

Genomic DNA sequences of all *SDW3* alleles described in this project are provided in Dataset S1. Raw transcriptomic data and expression values have been deposited in Gene Expression Omnibus (GSE282829) (32). Lists of differentially expressed genes in all nine pairwise comparisons are provided in separate tabs in Dataset S2. Raw GA quantification data have been deposited in Zenodo (https://zenodo.org/records/17910256) (30).
