# Supplementary material for: INDETERMINATE DOMAIN–DELLA protein interactions orchestrate gibberellin-mediated cell elongation in wheat and barley
Source: Proc Natl Acad Sci U S A. 2026 Jan 30;123(5):e2528934123. doi: 10.1073/pnas.2528934123 (PMC12867750; doi:10.1073/pnas.2528934123)
Supplement: Supplementary file 1 — Appendix 01 (PDF) [file pnas.2528934123.sapp.pdf]

INDETERMINATE DOMAIN-DELLA interactions orchestrate gibberellin-mediated cell elongation in wheat and barley

Figures S1 to S13
Tables S1 to S18

Figure S1. RNA-seq read coverage of wild-type 'Himalaya' and eight *sdw3* mutants across the genomic sequence of *HORVU.MOREX.r3.2HG0131300* (*SDW3*). Polymorphisms with the reference sequence are highlighted with stars. M671 (*sdw3a*) has a SNP in the intron 1 splice donor site causing aberrant splicing and a shift in read coverage across the first intron which is highlighted with a blue bar. Sequences of all alleles are provided in additional file 1.

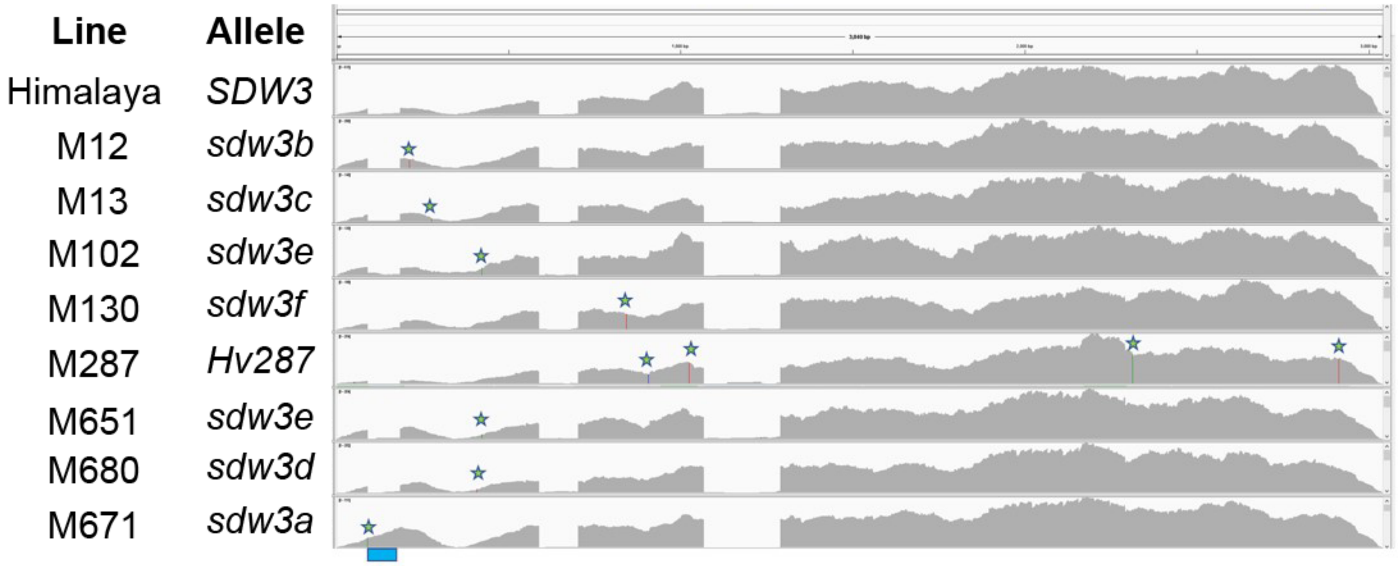

Figure S2. Phylogenetic tree of IDD proteins in Arabidopsis (red), rice (blue), barley (green), and wheat (black). Phylogenetic analysis was conducted using a BLOSUM62 substitution model with 1000 bootstraps and rooted to the *Marchantia polymorpha* IDD protein Mp5g20530 (purple). The ENY/GAF1 subclade is indicated.

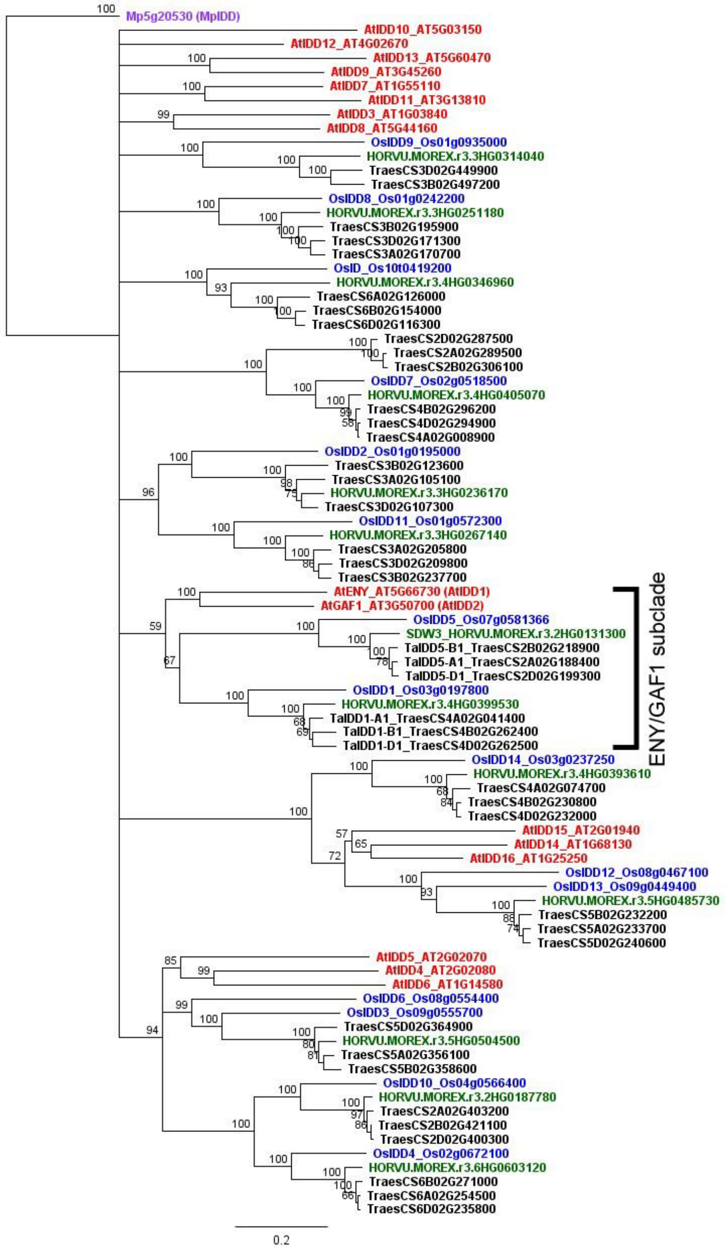

Figure S3. Amino acid alignment of Arabidopsis, wheat, barley and rice IDD proteins in the ENY/GAF1 subclade. The conserved ID domain, PAM domain and ERF motif are indicated. The position of *SDW3* alleles that encode non-synonymous amino acid substitutions and insertions are shown.

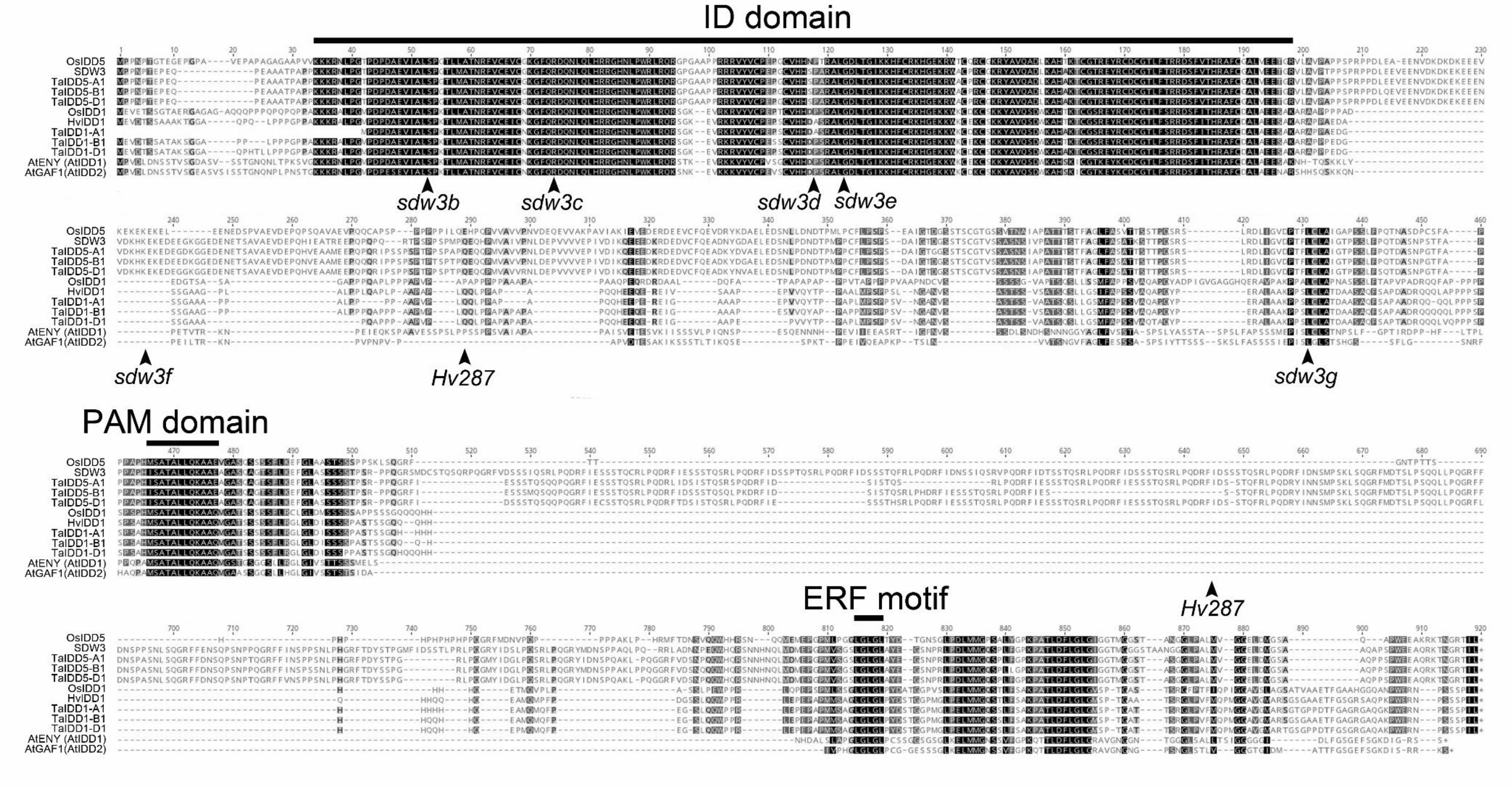

Figure S4. Pairwise amino acid identity (%) between full-length TaIDD5 and HvSDW3 protein sequences.

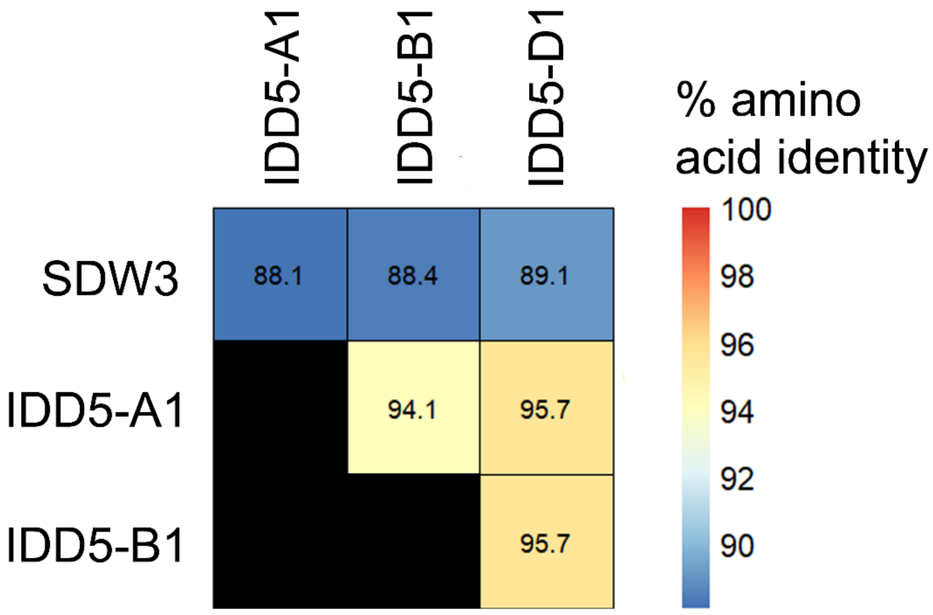

Figure S5. (A) Position of EMS-induced mutations in *IDD5-A1*, *IDD5-B1* and *IDD5-D1* used in this study. **(B)** Proportion of spliced and unspliced intron 1 reads in *IDD5-B1* in wild-type and CAD4-1415 lines. The proportion of reads mapping to each homoeologue was calculated manually from read pileups.

A

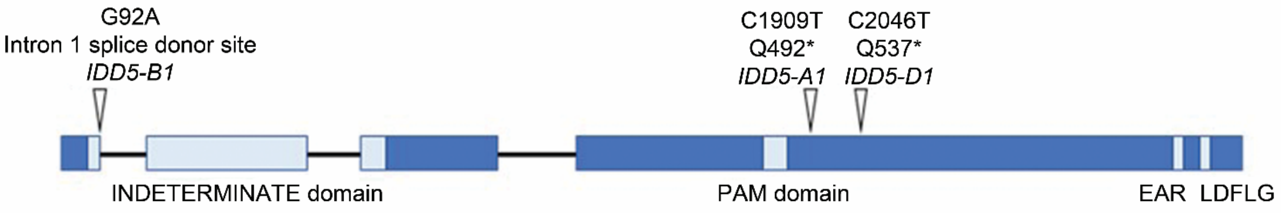

B

Genotype	% spliced reads	% unspliced reads	Contribution of homoeologues to unspliced reads	
Wild-type	92%	8%	*IDD5-A1*	28%
			IDD5-B1	40%
			IDD5-D1	32%
CAD4-1415	65%	35%	*IDD5-A1*	7%
			IDD5-B1	86%
			IDD5-D1	7%

Figure S6. GA dose response curve in L1 leaf blade tissues of seedlings 14 days after germination. Data were analysed using one-way ANOVA and Tukey's post-hoc HSD test.

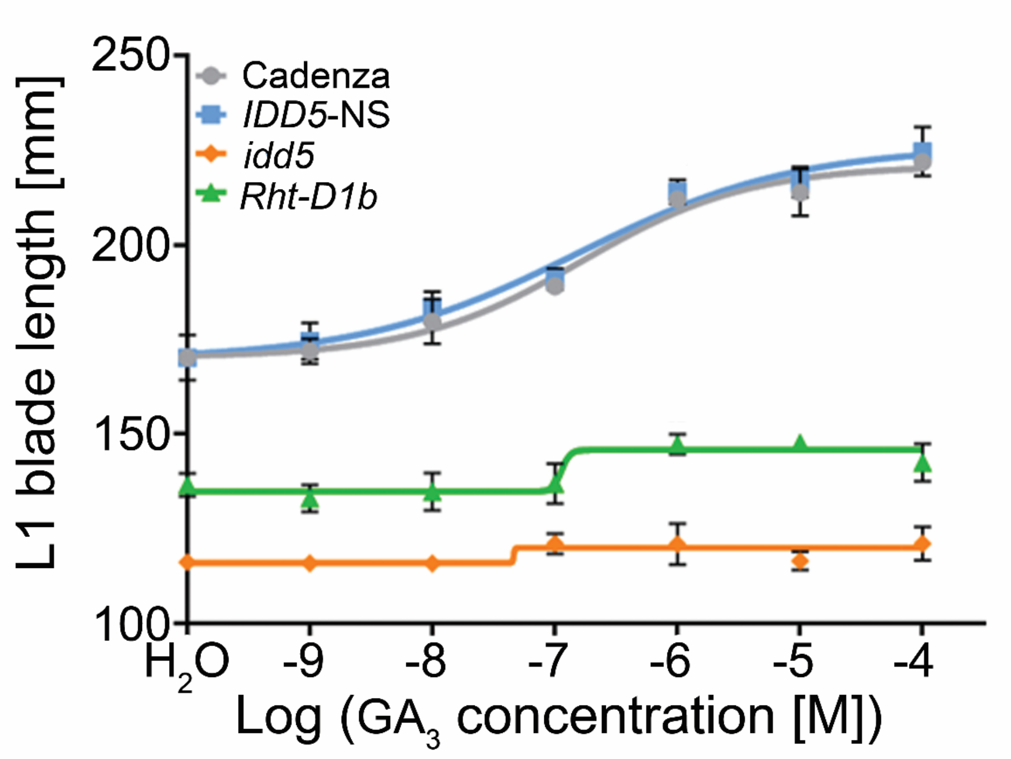

Figure S7. L1 sheath length (mm) of seven-day-old ‘Cadenza’ and *idd5* seedlings without (-GA) and after 100 µM GA_3 treatment (GA). Data were analysed with one-way ANOVA and Fishers post-hoc HSD test. Different letters indicate significant differences between genotypes at the 0.05 confidence level.

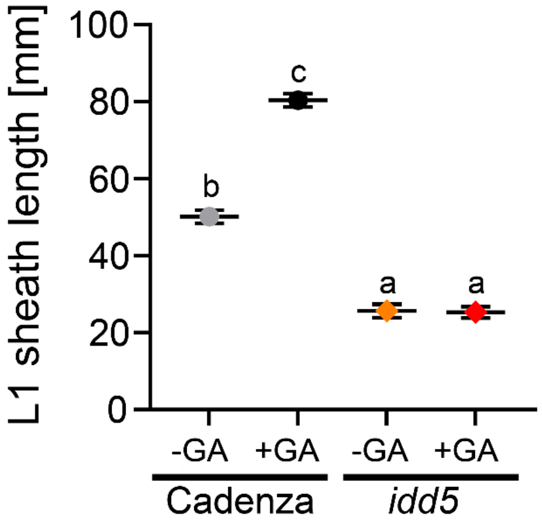

Figure S8. Principal Component Analysis plot of RNA-seq data generated from Cadenza, *Rht-D1b* and *idd5* lines.

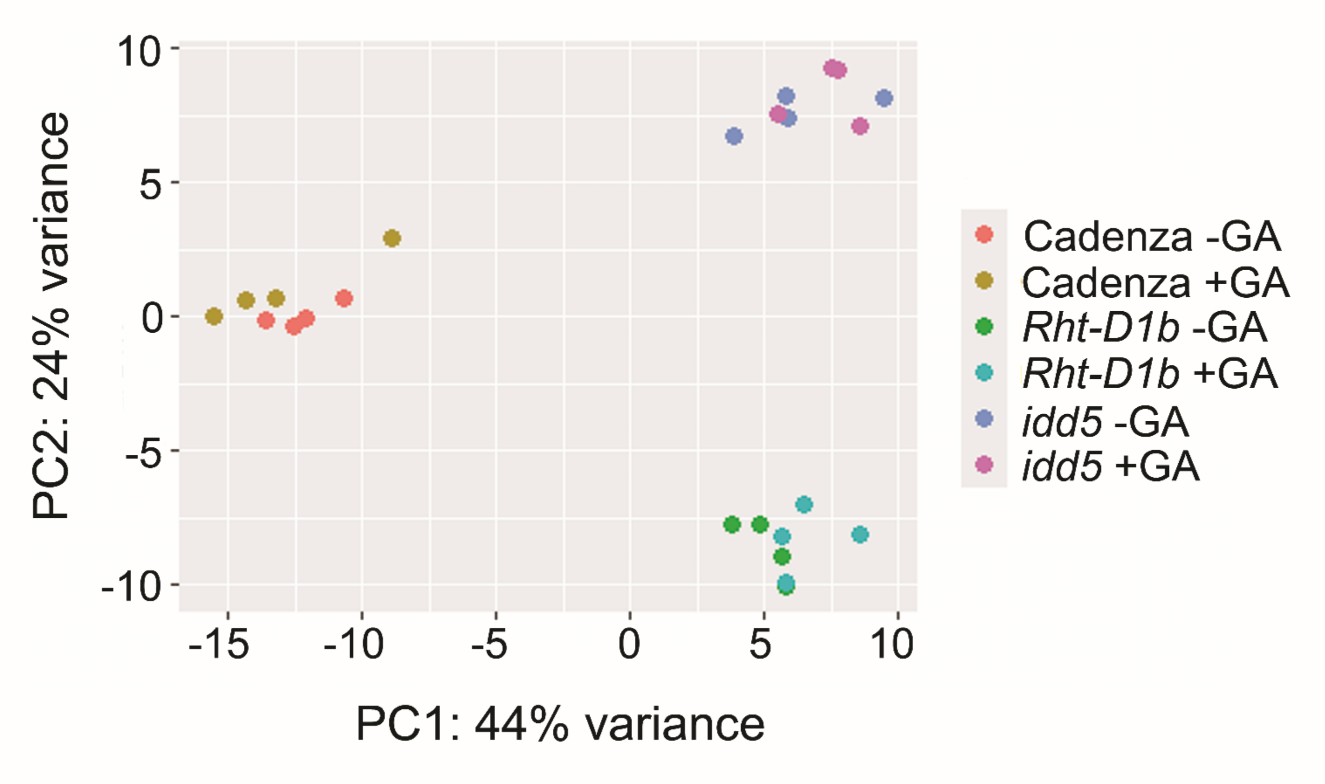

Figure S9. UpSet plot showing the number of DEGs in selected pairwise comparisons (P_{adj} < 0.01) and the subset of those genes that are common to different contrasts in GA-treated tissues of Cadenza, *Rht-D1b* and *idd5* genotypes.

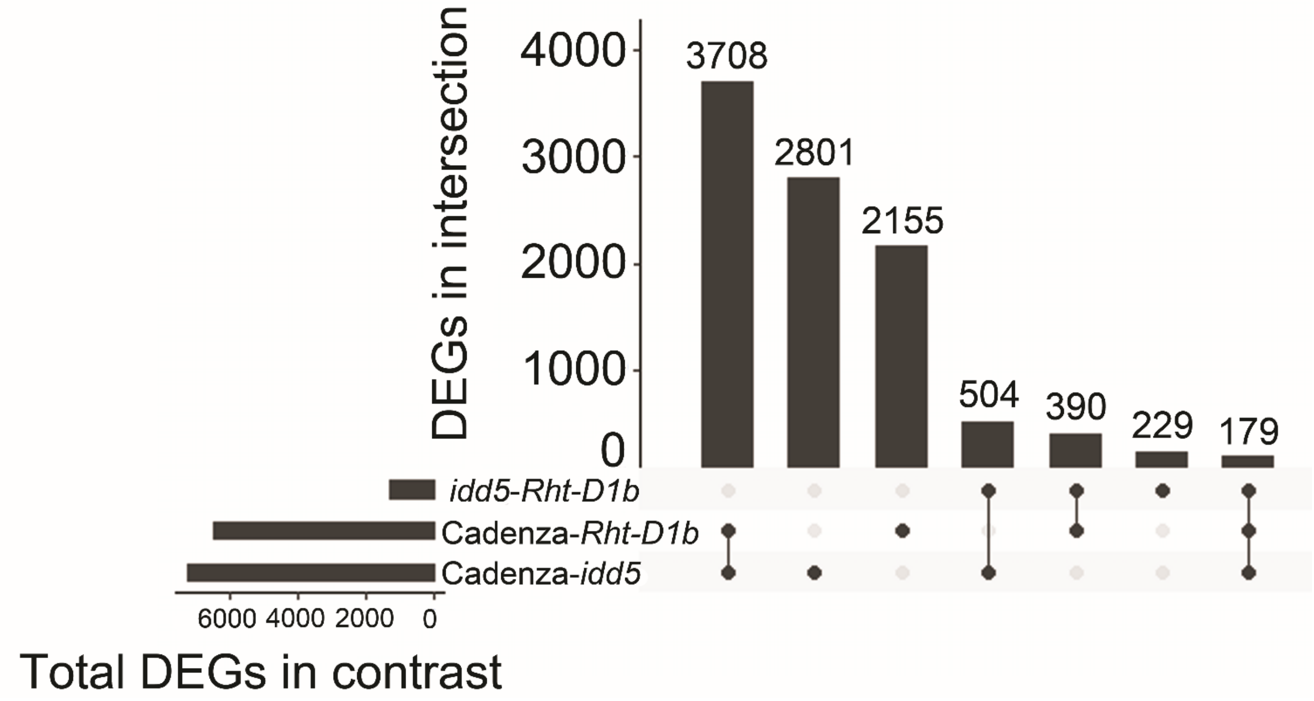

Figure S10. Representative images of the sub-crown internode (SCI) in *sdw3b* and *sdw3b/sln1c* double mutants.

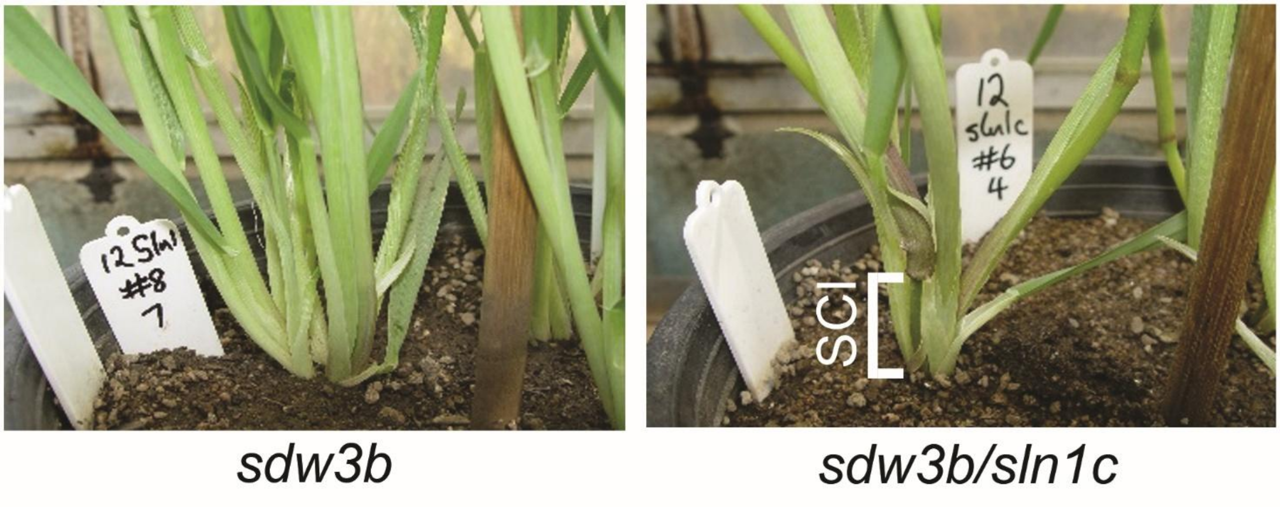

Figure S11. Stem length (mm) of single, double and triple *idd5* mutant genotypes in glasshouse conditions. Wild-type alleles are denoted in upper case and mutant alleles in lower case. Values are means ± SEM. Data were analysed with one-way ANOVA and Fishers post-hoc HSD test. Different letters indicate significant differences between genotypes at the 0.05 confidence level.

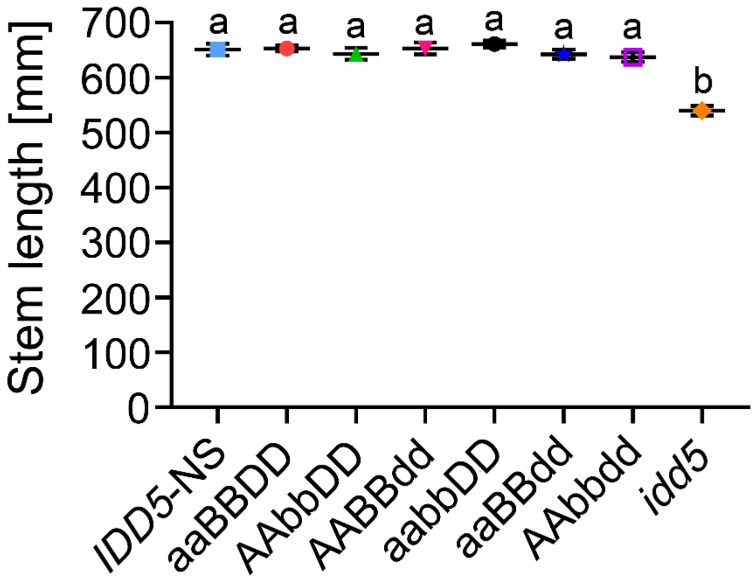

Figure S12. Length of different height components of Cadenza, *Rht-D1b* and *idd5* genotypes from the 2023 field experiment. P-1 = internode beneath the peduncle. The lengths of internodes P-3, P-4 and P-5 were combined for data analysis. Each section was measured from ten representative plants in each of four replicate blocks. Mean values ± SEM are shown. Data were analysed with linear mixed models fitted using restricted maximum likelihood. * indicates significant differences with Cadena at the 0.05 confidence level.

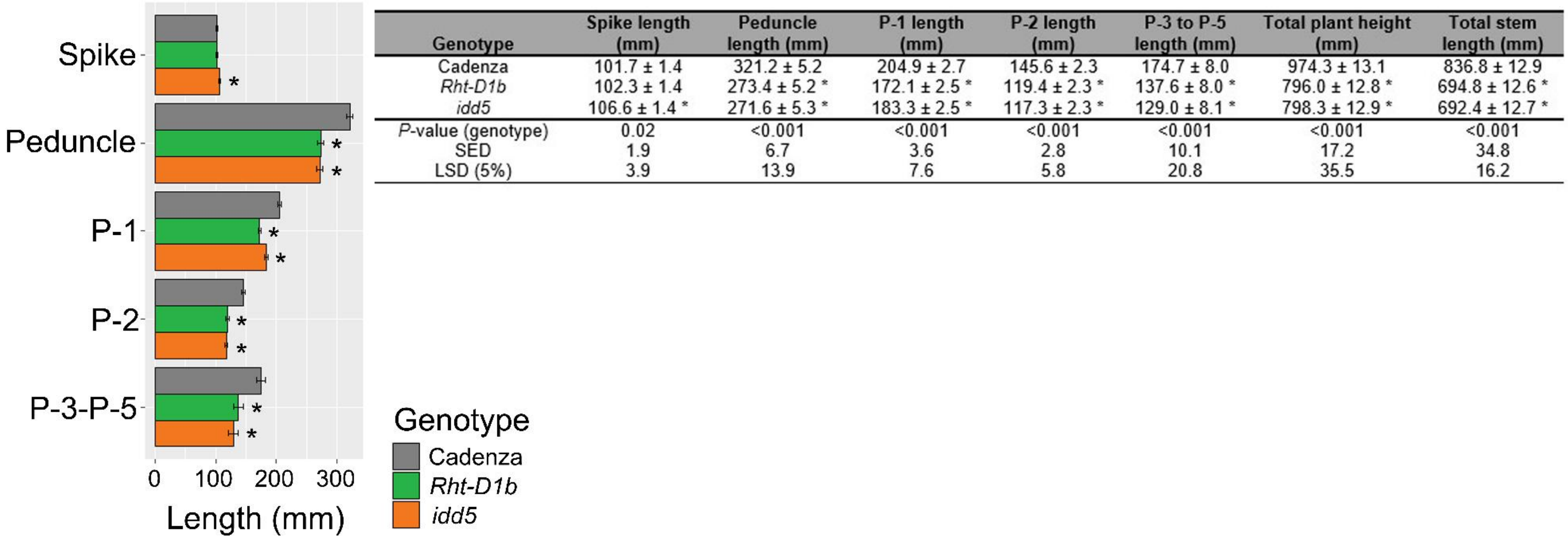

Genotype	Spike length (mm)	Peduncle length (mm)	P-1 length (mm)	P-2 length (mm)	P-3 to P-5 length (mm)	Total plant height (mm)	Total stem length (mm)
Cadenza	101.7 ± 1.4	321.2 ± 5.2	204.9 ± 2.7	145.6 ± 2.3	174.7 ± 8.0	974.3 ± 13.1	836.8 ± 12.9
Rht-D1b	102.3 ± 1.4	273.4 ± 5.2 *	172.1 ± 2.5 *	119.4 ± 2.3 *	137.6 ± 8.0 *	796.0 ± 12.8 *	694.8 ± 12.6 *
idd5	106.6 ± 1.4 *	271.6 ± 5.3 *	183.3 ± 2.5 *	117.3 ± 2.3 *	129.0 ± 8.1 *	798.3 ± 12.9 *	692.4 ± 12.7 *
P-value (genotype)	0.02	<0.001	<0.001	<0.001	<0.001	<0.001	<0.001
SED	1.9	6.7	3.6	2.8	10.1	17.2	34.8
LSD (5%)	3.9	13.9	7.6	5.8	20.8	35.5	16.2

Figure S13. Grains per spikelet in spike sections of Cadenza, *Rht-D1b* and *idd5* genotypes. Grains were counted manually from basal (spikelets 1-8), central (spikelets 9-17) and apical (spikelet 17 and above) for each genotype. Mean values ± SEM from four replicates are shown. Data were analysed with linear mixed models fitted using restricted maximum likelihood. For traits with non-significant genotype x trial interactions, joint effects are reported. * indicates significant differences with Cadena at the 0.05 confidence level.

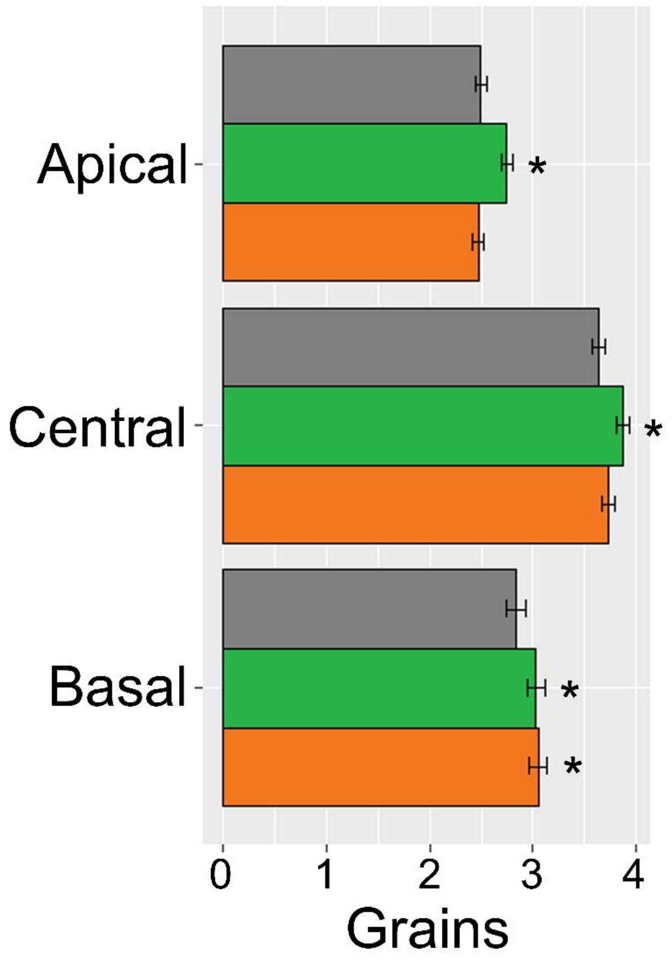

Trial	Genotype	Grains per spikelet		
		Basal (1-8)	Central (9-16)	Apical (17+)
**2022**	Cadenza	3.0 ± 0.1	3.6 ± 0.07	2.4 ± 0.06
	Rht-D1b	3.3 ± 0.1 *	3.9 ± 0.07 *	2.6 ± 0.06 *
	idd5	3.5 ± 0.1 *	3.8 ±0.07 *	2.4 ± 0.06
	P-value (genotype)	<0.001	0.002	0.003
	SED	0.1	0.08	0.07
	LSD (5%)	0.2	0.16	0.1
**2023**	Cadenza	2.7 ± 0.1	3.7 ± 0.1	2.6 ± 0.1
	Rht-D1b	2.8 ± 0.1	3.8 ± 0.1	2.9 ± 0.1
	idd5	2.6 ± 0.1	3.7 ± 0.1	2.5 ± 0.1
	P-value (genotype)	0.7	0.4	0.08
	SED	0.2	0.1	0.1
	LSD (5%)	0.5	0.3	0.3
**Combined**	Cadenza	2.8 ± 0.09	3.6 ± 0.07	2.5 ± 0.06
	Rht-D1b	3.0 ± 0.09	3.9 ± 0.07 *	2.7 ± 0.06 *
	idd5	3.1 ± 0.09	3.7 ± 0.07	2.5 ± 0.06
	P-value (genotype)	0.001	0.001	<0.001
	SED	0.1	0.08	0.08
	LSD (5%)	0.2	0.16	0.2
	P-value (genotype x trial)	0.08	0.7	0.4

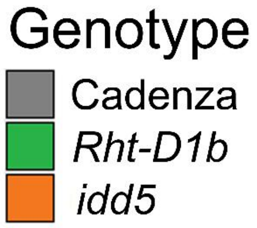

Supplemental tables

Table S1. Physical locations of *SDW3* markers on chromosome 2H of the Morex v2 and v3 barley genome assemblies. * = Previously identified gene-flanking markers described in Vu *et al*., 2010.

Marker	Position on chromosome 2H (bp)	
	Morex v2	Morex v3
TC146454	133,393,757	132,621,149
TC149567*	134,398,749	133,603,150
TC147542	136,535,222	135,675,412
TC144440	N/A	137,048,490
TC149841	139,015,090	138,038,964
SDW3 (*HORVU.MOREX.r3.2HG0131300*)	140,488,530	139,466,760
TC142185*	140,491,098	139,469,633
HW01K11(S2279)	141,321,401	140,285,297

Table S2. Maximum leaf extension rates (LER_{max}) in a Himalaya x M671 F_2 population segregating for the *sdw3a* allele. Values are means ± SEM. Data was analysed using unbalanced two-way ANOVA. *P*-values represent the contrast between control and GA-treated seedlings from each genotype.

	**LER_{max} (mm d^{-1})**		
**Genotype**	**Control**	**+ 10 µM GA_3**	***P*-value**
Himalaya (*SDW3*/*SDW3*)	28.5 ± 1.2 (*N* = 10)	51.7 ± 2.3 (*N* = 3)	< 0.001
Heterozygote (*SDW3*/*sdw3a*)	27.9 ± 0.7 (*N* = 28)	36.2 ± 0.8 (*N* = 27)	< 0.001
M671 (*sdw3a*/*sdw3a*)	21.3 ± 1.2 (*N* = 10)	21.7 ± 1.1 (*N* = 13)	0.812

Table S3. Maximum leaf extension (LER_{max}) rates in Himalaya, *gse1a* and *sdw3a* genotypes in response to treatment with low (10 μM) and high (10 mM) GA_3 concentrations. Values are means ± SEM *N* = 8.

Genotype	LER_{max} (mm d^{-1})		
	Control	10 uM GA_3	10 mM GA_3
Himalaya	28.3 ± 6.0	58.4 ± 1.2	62.7 ± 1.4
gse1a	9.7 ± 0.4	22.0 ± 0.6	42.1 ± 0.9
sdw3a	18.7 ± 0.5	19.5 ± 0.3	19.9 ± 0.3

Table S4. Maximum leaf extension rates (LER_{max}) in Himalaya, *grd2b, sdw3a* and *sdw3b* genotypes in response to 10 μM PBZ and 10 μM PBZ + 10 μM GA_3 treatments. Values are means ± SEM *N* = 8.

**Genotype**	**LER_{max} (mm d^{-1})**		
	**Control**	**PBZ**	**PBZ + GA_3**
Himalaya	48.3 ± 1.1	25.2 ± 1.4	60.9 ± 0.9
grd2b	26.9 ± 2.2	19.5 ± 0.7	56.3 ± 2.3
sdw3a	24.1 ± 0.9	13.6 ± 1.1	22.5 ± 0.7
sdw3b	30.6 ± 1.7	9.3 ± 2.3	23.9 ± 1.9

Table S5. L1 leaf sheath length (mm) in Cadenza, *IDD5*-NS, *idd5* and *Rht-D1b* genotypes. Values are means ± SD. Data were analysed with one-way ANOVA and Tukey's post-hoc HSD test. Different letters indicate significant differences between genotypes at the 0.05 confidence level.

Genotype	L1 leaf sheath length (mm)
Cadenza	62.5 ± 3.8 a
IDD5-NS	62.4 ± 6.4 a
idd5	42.1 ± 4.7 b
Rht-D1b	40.0 ± 7.8 b
P-value (genotype)	<0.001
LSD at 5% (mm)	3.37

Table S6. GA dose-response assays for L1 leaf sheath tissues in Cadenza, IDD5-NS, *idd5* and *Rht-D1b* genotypes. Values are means. Data were analysed with one-way ANOVA and Tukey's post-hoc HSD test. Different letters indicate significant differences between genotypes at the 0.05 confidence level.

**Genotype**	**H_2O**	**10^{-9} M GA_3**	**10^{-8} M GA_3**	**10^{-7} M GA_3**	**10^{-6} M GA_3**	**10^{-5} M GA_3**	**10^{-4} M GA_3**
Cadenza	62.5 a	63.6 a	67.0 a	82.8 a	103.5 a	111.2 a	106.1 a
IDD5-NS	62.4 a	61.4 a	65.9 a	81.7 a	99.8 a	107.8 a	103.5 a
idd5	42.0 b	41.7 b	43.1 b	44.1 b	44.3 b	47.1 b	45.7 b
Rht-D1b	40.0 b	37.3 c	42.6 b	44.7 b	43.6 b	48.1 b	40.8 b
P-value (genotype)	<0.001	<0.001	<0.001	<0.001	<0.001	<0.001	<0.001
SED	1.694	1.552	1.669	1.982	2.061	2.271	2.325
LSD 5% (mm)	3.365	3.085	3.317	3.938	4.096	4.515	4.618

Table S7. GA dose-response assays for L1 leaf blade tissues in Cadenza, IDD5-NS, *idd5* and *Rht-D1b* genotypes. Data were analysed with one-way ANOVA and Tukey's post-hoc HSD test. Different letters indicate significant differences between genotypes at the 0.05 confidence level.

**Genotype**	**H_2O**	**10^{-9} M GA**	**10^{-8} M GA**	**10^{-7} M GA**	**10^{-6} M GA**	**10^{-5} M GA**	**10^{-4} M GA**
Cadenza	172.8 a	171.9 a	179.7 a	189.1 a	212.0 a	213.8 a	221.9 a
IDD5-NS	170.2 a	172.9 a	183.6 a	191.0 a	214.0 a	216.6 a	224.7 a
idd5	116.2 b	115.9 b	115.9 b	121.1 b	121.0 b	116.5 b	121.1 b
Rht-D1b	136.6 c	135.1 c	134.9 c	136.8 c	147.4 c	147.7 c	144.6 c
P-value (genotype)	<0.001	<0.001	<0.001	<0.001	<0.001	<0.001	<0.001
SED	2.81	2.95	2.93	2.86	3.13	3.06	3.47
LSD 5% (mm)	5.59	5.87	5.82	5.69	6.22	6.08	6.92

Table S8. Abaxial cell lengths in 'Cadenza' and *idd5* genotypes in response to GA_3. Values are means ± SEM. Data were analysed using two-way ANOVA as a 2 x 2 factorial.

Genotype	Abaxial cell length (μm)	
	**Control**	**+ 10 μM GA_3**
Cadenza	1005.4 ± 13.6	1137.8 ± 17.1
idd5	822.3 ± 10.3	850.3 ± 11.5
Genotype	F (1,82.6) = 119.1, P < 0.001	
Treatment	F (1,79.2) = 12.1, P < 0.001	
Genotype x treatment	F (1,84.6) = 5.8, P = 0.018	

Table S9. GA levels in leaf sheath tissues of 7-day-old seedlings from Cadenza, *Rht-D1b*, *IDD5*-NS and *idd5* genotypes. Values are mean GA levels (pg/mg) on a dry weight basis ± SEM. ND = not detected. Data was analysed by one-way ANOVA and Tukey's post-hoc HSD test.

	Cadenza	*Rht-D1b*	*IDD5*-NS	*idd5*	*P*-value (genotype)
GA_{15}	ND	ND	ND	ND	-
GA_{24}	ND	ND	ND	ND	-
GA_{9}	ND	ND	ND	ND	-
GA_{4}	ND	0.261 ± 0.218	ND	ND	-
GA_{34}	0.030 ± 0.004	0.025 ± 0.011	0.022 ± 0.005	0.022 ± 0.002	0.141
GA_{7}	ND	ND	ND	ND	-
GA_{54}	ND	ND	ND	ND	-
GA_{61}	ND	ND	ND	ND	-
GA_{53}	0.013 ± 0.007	ND	0.014 ± 0.007	0.008 ± 0.003	0.224
GA_{44}	2.282 ± 0.442	0.632 ± 0.152	2.364 ± 0.308	0.396 ± 0.015	1.62E-07
GA_{19}	1.079 ± 0.175	0.505 ± 0.145	0.794 ± 0.216	0.366 ± 0.049	2.73E-06
GA_{20}	0.613 ± 0.037	0.817 ± 0.085	0.974 ± 0.092	0.477 ± 0.205	0.0272
GA_{1}	1.027 ± 0.170	2.810 ± 0.413	0.290 ± 0.092	1.933 ± 0.071	4.14E-07
GA_{29}	0.407 ± 0.064	0.084 ± 0.035	0.290 ± 0.063	0.147 ± 0.035	7.28E-05
GA_{8}	3.174 ± 0.534	2.573 ± 0.317	4.120 ± 0.447	4.084 ± 0.385	0.00256
GA_{3}	0.274 ± 0.087	0.394 ± 0.058	0.330 ± 0.185	0.305 ± 0.043	0.441
GA_{51}	ND	ND	ND	ND	-

Table S10. Plant height, stem length, spike length and spikelet number of wild-type, *rht1*, *idd5* and *rht1/idd5* mutants grown in glasshouse conditions. Values are mean ± SEM. Data were analysed with one-way ANOVA and Tukey's post-hoc HSD test. Different letters indicate significant differences between genotypes at the 0.05 confidence level.

Genotype	Plant height (mm)	Stem length (mm)	Spike length (mm)	Spikelet number
Cadenza	749.1 ± 3.2 a	642.1 ± 15.4 a	107.0 ± 1.3 a	19.5 ± 0.3 a
rht1	1107.4 ± 20.9 b	953.0 ± 89.8 b	154.4 ± 3.8 b	14.5 ± 0.3 b
idd5	643.6 ± 4.1 c	542.1 ± 18.4 c	104.4 ± 1.5 a	18.7 ± 0.3 a
rht1/idd5	635.3 ± 6.0 c	521.2 ± 22.6 c	114.1 ± 1.8 c	19.1 ± 0.2 a
P-value (genotype)	<0.001	<0.001	<0.001	<0.001
SED	16.49	16.97	3.29	0.525
LSD at 5%	35.15	36.16	7.02	1.118

Table S11. Plant height of Himalaya wild-type, *sdw3a* and *sdw3b* genotypes in a wild-type and *sln1c* background grown in glasshouse conditions. Values are means ± SEM. Data was analysed with one-way ANOVA and Tukey's HSD post hoc test. Different letters indicate significant differences between genotypic classes at the 0.05 confidence level.

Genotype	Height (cm)
Himalaya	88.9 ± 1.1 a
sdw3b	79.6 ± 1.7 b
sdw3b/sln1c	93.0 ± 1.5 a
sdw3a	66.7 ± 1.4 c
sdw3a/sln1c	69.1 ± 1.8 c
P-value (genotype)	<0.001
SED	2.173
LSD at 5%	4.359

Table S12. Plant height of Himalaya wild-type, *sdw3a*, *sdw3b* and *sdw3e* genotypes in glasshouse conditions. Values are means ± SEM. Data was analysed with one-way ANOVA and Tukey's HSD post hoc test. Different letters indicate significant differences between genotypic classes at the 0.05 confidence level.

Genotype	Height (cm)
Himalaya	88.7 ± 1.2 a
sdw3b	79.6 ± 1.7 b
sdw3e	68.6 ± 1.4 c
sdw3a	66.7 ± 1.4 c
P-value (genotype)	<0.001
SED	2.022
LSD at 5%	4.083

Table S13. Phenotypic data of single, double and triple *idd5* mutant genotypes in glasshouse conditions. Wild-type alleles are denoted in upper case and mutant alleles in lower case. Values are means ± SEM. Data were analysed with one-way ANOVA and Fishers post-hoc HSD test. Different letters indicate significant differences between genotypes at the 0.05 confidence level.

Genotype	Stem length (mm)	Peduncle length (mm)	Ear length (mm)	Spikelet number
Cadenza	674.7 ± 9.9 a	351.7 ± 9.7 c	90.7 ± 2.0 a	17.4 ± 0.7 ab
IDD5-NS	651 ± 11.1 a	325.1 ± 14.9 bc	95.4 ± 1.8 a	17.6 ± 0.6 ab
aaBBDD	653.2 ± 5.3 a	320.5 ± 10.5 abc	95.6 ± 2.0 a	18.8 ± 0.6 ab
AAbbDD	643.3 ± 10.6 a	337.1 ± 11.0bc	91.5 ± 3.3 a	16.2 ± 0.7 a
AABBdd	653.3 ± 10.9 a	349.0 ± 9.4 c	98.0 ± 2.3 a	16.8 ± 0.5 ab
aabbDD	661.2 ± 6.7 a	296.9 ± 6.2 ab	98.2 ± 2.1 a	19.4 ± 0.3 ab
aaBBdd	645.4 ± 8.6a	328.8 ± 12.8 bc	98.9 ± 1.8 a	18.3 ± 0.7 ab
AAbbdd	637.5 ± 8.8a	317.3 ± 9.8 abc	98.8 ± 1.5 a	18.3 ± 0.5 ab
idd5	539.7 ± 8.7 b	273.3 ± 9.0 a	97.3 ± 1.9 a	18.7 ± 0.7 b
P-value (genotype)	<.001	<.001	0.109	0.021
SED	19.60	14.65	3.165	0.846
LSD at 5%	40.54	30.30	6.547	1.750

Table S14. Stem length phenotypes of *Rht-D1b* and *idd5* genotypes with their appropriate control lines in glasshouse conditions. Values are means ± SEM. Data were analysed using one-way ANOVA. The values significantly different between Cadenza and *IDD5*-NS are denoted by "*" and values that are significantly different between *idd5* and *Rht-D1b* are denoted by "^".

Genotype	Stem length (mm)	Peduncle length (mm)	P-1 length (mm)	P-2 length (mm)	P-3 length (mm)
Cadenza	686.3 ± 35.6	330.2 ± 46.0	171.8 ± 16.4	112.0 ± 13.5	62.4 ± 24.3
IDD5-NS	693.8 ± 38.6	344.6 ± 34.5	173.3 ± 13.6	113.8 ± 12.1	63.4 ± 21.6
idd5	544.6 ± 29.1***^**	275.6 ± 30.8*****	145.2 ± 14.7***^**	81.4 ± 11.9***^**	40.7 ± 15.3***^**
Rht-D1b	508.5 ± 22.2*****	287.0 ± 24.8*****	133.2 ± 12.7*****	66.2 ± 14.2*****	22.0 ± 14.6*****
P-value (df = 69)	<0.001	<0.001	<0.001	<0.001	<0.001
LSD at 5% [mm]	15.7	19.5	8	7.3	11.4

Table S15. Coleoptile and root length in ten-day-old seedlings of Cadenza, *idd5* and *Rht-D1b* genotypes. Values are means ± SEM from seedlings germinated from 100 grains. *** $P < 0.001$ from two-tailed Student's t-test with Cadenza.

Genotype	Coleoptile length (mm)	Root length (mm)	Germination rate (%)
Cadenza	133.1 ± 3.9	168.5 ± 3.1	88
Rht-D1b	106.5 ± 2.4***	163.4 ± 2.6	92
idd5	102.0 ± 3.6***	160.3 ± 3.7	92

Table S16. Phenotypes of ‘Cadenza’, *Rht-D1b* and *idd5* genotypes in 2022 and 2023 field experiments. Values are means ± SEM. Data were analysed with linear mixed models fitted using restricted maximum likelihood. * indicates significant differences with Cadena at the 0.05 confidence level. For traits with non-significant genotype x trial interactions, joint effects are reported.

Trial	Genotype	Plot height (cm)	Plot yield (t/ha)	Spikelet number	Grains per spike	TGW (g)	Grain weight per spike (g)	Spike number (/ha)
**2022**	Cadenza	91.9 ± 1.2	10.2 ± 0.5	23.2 ± 0.3	69.5 ± 2.0	50.9 ± 0.6	3.5 ± 0.09	287.5 ± 15.7
	Rht-D1b	79.0 ± 1.2 *	10.4 ± 0.5	23.1 ± 0.3	75.9 ± 2.0	51.1 ± 0.6	3.9 ± 0.09 *	263.7 ± 15.7
	idd5	80.8 ± 1.2 *	9.0 ± 0.5 *	24.4 ± 0.3 *	78.4 ± 2.0	48.9 ± 0.6 *	3.8 ± 0.09	234.5 ± 15.7 *
	P-value (genotype)	<0.001	<0.001	<0.001	<0.001	0.003	0.005	0.002
	SED	1.2	0.4	0.3	2.0	0.7	0.1	14.3
	LSD (5%)	2.4	0.7	0.5	4.0	1.4	0.3	28.5
**2023**	Cadenza	104.1 ± 1.0	9.7 ± 0.5	24.0 ± 0.2	72.1 ± 2.8	37.5 ± 0.4	2.7 ± 0.2	360.1 ± 23.9
	Rht-D1b	89.8 ± 1.0 *	9.6 ± 0.5	24.7 ± 0.2	78.3± 2.8	36.2 ± 0.4	2.8 ± 0.2	342.6 ± 23.9
	idd5	84.5 ± 1.0 *	8.3 ± 0.5 *	25.8 ± 0.2 *	75.3 ± 2.8	39.5 ± 0.4	3.4 ± 0.2	250.4 ± 23.9 *
	P-value (genotype)	<0.001	0.04	<0.001	0.33	<0.001	<0.001	0.02
	SED	1.5	0.7	0.2	3.9	0.3	0.3	33.8
	LSD (5%)	2.9	1.4	0.5	8.9	0.8	0.9	76.4
**Combined**	Cadenza	-	10.0 ± 0.3	23.6 ± 0.2	70.8 ± 1.7	-	3.1 ± 0.1	323.8 ± 14.3
	Rht-D1b	-	10.0 ± 0.3	24.0 ± 0.2	77.1 ± 1.7 *	-	3.4 ± 0.1	303.2 ± 14.3
	idd5	-	8.7 ± 0.3 *	25.1 ± 0.2	76.8 ± 1.7 *	-	3.6 ± 0.1 *	242.5 ± 14.3 *
	P-value (genotype)	-	<0.001	<0.001	<0.001	-	<0.001	<0.001
	SED	-	0.4	0.2	2.2	-	0.2	18.3
	LSD (5%)	-	0.8	0.4	4.4	-	0.4	36.4
	P-value (genotype x trial)	<0.001	0.9	0.10	0.37	<0.001	0.22	0.21

Table S17. SDW3 proteins in 44 barley wheat accessions. The presence of the 15 amino acid insertion described in line Hv287 is indicated.

Accession	SDW3 protein ID	15 amino acid insertion
10tj18	HORVU.10tj18.PROJ.2HG00087530.1	
Aizu	HORVU.AIZU_6.PROJ.2HG00087570.1	
Akashinriki	HORVU.AKASHINRIKI.PROJ.2HG00119510.1	
B1k-17-07	HORVU.B1K-17-07.PROJ.2HG00084530.1	Yes
B1k-33-13	HORVU.B1K-33-13.PROJ.2HG00084920.1	
Barke	HORVU.BARKE.PROJ.2HG00120340.1	
Bonus	HORVU.BONUS.PROJ.2HG00088590.1	
Bowman	HORVU.BOWMAN.PROJ.2HG00088620.1	
F2327	HORVU.F2327.PROJ.2HG00084260.1	Yes
Foma	HORVU.FOMA.PROJ.2HG00089360.1	
HID12xxi	HORVU.HID12XXI.PROJ.2HG00084220.1	
HID251	HORVU.HID251.PROJ.2HG00085320.1	
HID84	HORVU.HID84.PROJ.2HG00084180.1	
HOR_10350	HORVU.HOR_10350.PROJ.2HG00114470.1	Yes
HOR_1168	HORVU.HOR_1168.PROJ.2HG00087760.1	Yes
HOR_12184	HORVU.HOR_12184.PROJ.2HG00086820.1	
HOR_13594	HORVU.HOR_13594.PROJ.2HG00087140.1	Yes
HOR_13663	HORVU.HOR_13663.PROJ.2HG00088400.1	
HOR_13942	HORVU.HOR_13942.PROJ.2HG00117300.1	Yes
HOR_14061	HORVU.HOR_14061.PROJ.2HG00088400.1	Yes
HOR_14121	HORVU.HOR_14121.PROJ.2HG00087960.1	Yes
HOR_1702	HORVU.HOR_1702.PROJ.2HG00087910.1	Yes
HOR_18321	HORVU.HOR_18321.PROJ.2HG00089990.1	Yes
HOR_21322	HORVU.HOR_21322.PROJ.2HG00088160.1	Yes
HOR_3365	HORVU.HOR_3365.PROJ.2HG00117430.1	Yes
HOR_4224	HORVU.HOR_4224.PROJ.2HG00087910.1	
HOR_495	HORVU.HOR_495.PROJ.2HG00086980.1	
HOR_6220	HORVU.HOR_6220.PROJ.2HG00088220.1	Yes
HOR_7385	HORVU.HOR_7385.PROJ.2HG00086820.1	
HOR_7552	HORVU.HOR_7552.PROJ.2HG00118970.1	
HOR_9043	HORVU.HOR_9043.PROJ.2HG00118950.1	Yes
Chikurin Ibaraki	HORVU.CHIKURIN_IBARAKI.PROJ.2HG00088340.1	
Igri	HORVU.IGRI.PROJ.2HG00117850.1	
Golden Melon	HORVU.GOLDEN_MELON.PROJ.2HG00087590.1	
Morex	HORVU.MOREX.PROJ.2HG00116160.1	
OUN333	HORVU.OUN333.PROJ.2HG00119950.1	
RGT Planet	HORVU.RGT_PLANET.PROJ.2HG00118280.1	
WBDC_103	HORVU.WBDC103.PROJ.2HG00084050.1	

WBDC_199	HORVU.WBDC199.PROJ.2HG00084890.1	Yes
WBDC_207	HORVU.WBDC207.PROJ.2HG00085760.1	Yes
WBDC_237	HORVU.WBDC237.PROJ.2HG00085260.1	Yes
WBDC_348	HORVU.WBDC348.PROJ.2HG00084250.1	Yes
ZDM01467	HORVU.ZDM01467.PROJ.2HG00116840.1	
ZDM02064	HORVU.ZDM02064.PROJ.2HG00118750.1	

Table S18. Sequences of primers used in this study. CAPS markers include the expected sizes of digested amplicons to distinguish genotypes.

Oligo name	Sequence (5' - 3')	Purpose	Details
Sdw3_F1	GGAGAGAGAGGGAGGGAAAA	*SDW3* genotyping	
Sdw3_R1	CCACGGCAGAAGTCTCATTT		
Sdw3_F2	CACATCGCGCTTTCTGTG		
Sdw3_R2	GCCGTTGGGATTGTGTTG		
Sdw3_F3	GGCACACCATCCTCTCTGTT		
Sdw3_R3	TTTTCCTACCACCACCCAAC		
BC1_NGS_BS_FOR	CCATCTCATCCCTGCGTGTCTCCGACTCAGCT AAGGTAACGATCGCCGCCCAAGAAGAAGAGG	*IDD5* amplicon sequencing to assess splicing variation in *IDD5-B1*	
BC2_NGS_BS_FOR	CCATCTCATCCCTGCGTGTCTCCGACTCAGTA AGGAGAACGATCGCCGCCCAAGAAGAAGAGG		
NGS_CR_BS_REV	CCTCTCTATGGGCAGTCGGTGATGCTCCGCA CACGAACCGGTTGGTC		
IDD5-A_F	GTACACCATCATCTCTGTTCCCA	CAPS marker to detect *IDD5-A1* mutation	*Sty*I digest<br>WT = 644/237/45 bp<br>MT = 644/282 bp
IDD5-A_R	GCCTGCGGTGAGTTGTCG		
IDD5-B_WT_FAM	GAAGGTGACCAAGTTCATGCTCTCCCCGGGACGCCAGG	KASP marker to genotype *IDD5-B1*	
IDD5-B_MUT_HEX	GAAGGTCGGAGTCAACGGATTCTCCCCGGGACGCCAGA		
IDD5-B_CR	GCAAAACCCGAAGCACGCGG		
IDD5-D_WT_FAM	GAAGGTGACCAAGTTCATGCTACACAATCCCGGTTACCCC	KASP marker to genotype *IDD5-D1*	
IDD5-D_MUT_HEX	GAAGGTCGGAGTCAACGGATTACACAATCCCGGTTACCCT		
IDD5-D_CR	AACCGGGAATGTGTTGAGC		
Rht-1F3_generic	CAGATCTGCAACGTGGTGG	CAPS marker to detect *RHT-A1* mutation	*Nar*I digest<br>WT = 246/135 bp<br>MT = 381 bp
Rht-A1R3	CATTTAGCTTCTTCTTCAGAGG		
Rht-1F3_generic	CAGATCTGCAACGTGGTGG	CAPS marker to detect *RHT-D1* mutation	*Tsp*45I digest<br>WT = 451 bp<br>MT = 386bp/65 bp
Rht-D1R3	CGGAACCACCCGTAGCCCGA		
