# Supplementary material for: INDETERMINATE DOMAIN–DELLA protein interactions orchestrate gibberellin-mediated cell elongation in wheat and barley
Source: Proc Natl Acad Sci U S A. 2026 Jan 30;123(5):e2528934123. doi: 10.1073/pnas.2528934123 (PMC12867750; doi:10.1073/pnas.2528934123)
Supplement: Supplementary file 2 — Dataset S01 (PDF) [file pnas.2528934123.sd01.pdf]

>SDW3_Himalaya_wild-type_gDNA

ATGCCGCCCAATCCGACGGAGCCGGAGCAGCCGGAGGCGGCCGCGACGCCGGCGCCGCCCAAGA
AGAAGAGGAACCTCCCCGGGACGCCAGGCGAGAGCGCGTGCTTCGGATTTTGCGGGGTTGTTGGTAC
GGCTCGCGCGAGCTTTCTTAGTCTGACGGTGGTCGGTTTCCGTGTGCTTGTGCAGATCCGGACGCGGAG
GTGATCGCGCTGTCGCCGGGGACGCTCATGGCGACCAACCGGTTCGTGTGCGAGGTGTGCGGCAAGG
GCTTCCAGAGGGACCAGAACCTGCAGCTGCACCGCCGGGGGCACAACCTTCCGTGGCGGCTGCGG
CAGCGCGGCCCCGGGGCGGCGCCGCCGCGCCGGAGGGTCTACGTCTGCCCGGAGCCCGGCTGC
GTGCACCACTCCCCCGCCCGCGCGCTCGGGGACCTCACGGGTATCAAGAAGCACTTTTGCCGCAAG
CACGGCGAGAAGCGATGGGCCTGCCCACGCTGCGGCAAGCGCTACGCCGTCCAGGCCGACCTCAA
GGCCCATGCCAAGACCTGCGGCACCCGCGAGTATCGCTGTGACTGCGGCACGCTCTTCACCAGGTAC
TACTTCCAGCTCGGTTCCCCCCAATTTCGATGGCTCCTTTTTCCTTCCCTTGAGGGACAAATGTTTGTACTT
TTGTTTCATTCATCAGTCGGCGTGCTGGATTGATTCAGGAGAGACAGTTTCGTGACACATCGCGCTTTCTGT
GGCGCTCTCGTCGAGGAGACAGGCAGGGTGCTTGCTGTTCCGACGCCGCCTTCGCCTCGGCCACCTG
ATTTGGAGGAGGTTGAGGAGAATGTAGACAAGGACAAGGAAAAAGAAGAGGAGAATGTGGATAAGCACAA
GGAGAAGGAAGATGAGGAGGGCAAGGGGGGAGAAGATGAAAATGAGACTTCTGCCGTGGCCGAGGTG
GATGAGCCGCAGCACATTGAGGCAACAAGGGAGGAGCCACAGCCACAGCCACAGCGGACTCCGTCG
CCGCCATCTCCAATGCCACAGGAGCAGCACCCAATGGTGGCAATTGTGCCAAATTTGGATGGTATGCTT
CTTTTGCATTTTGCAATGTGTTTGTCTTACTACCTATTCATAGACCATGTTAGATTTATAGCAAACTCATTCTCC
CTAATCCATCTTTATTTCTTATCACTAGCAAAAAAAGGAATGCATCAGTCTTGTTTCTTTCCCAATTTAGCTAG
CCTTATGATCATTCAGTCAACAATGATGAGTGACCTGTTCTTTTGTACCATGCCTATATGTTACAGAGCCAGT
GGTGGTTGTGGAGCCAATTGTGGATATCAAGCAAGAGGAGGAAGATAAGCGAGATGAAGATGTTTGCTTC
CAGGAAGCAGATAATTACGGCGATGCTGAACTAGAAGACTCCAACTTGCCAGATAATGATACCCCGATGC
CTCCTTGTTTCCTCCCATCGCCCTCGGATGCCATTGGTACAGATGGCAGCAGCACCAGTTGTGGCACAG
TCAGCAGCGCATCCAATTCCATCGTGCCAGCAACGACGACTAGCACATTTGCGGGGCTGTTTGCATCAG
CCACAAAAAGCACCACTCCCCAGAGCAGATCGCTGCGTGATCTTATCGGTGTTGATCCCACCTTCCTTTG
CCTTGCAATCGGCACACCATCCTCTCTGTTCCCGCAGACAGATGCAAGCAACCCCAGCACCTTTGCTCC
ACCTCCAGCACCACACATCTCCGCAACTGCGCTCCTGCAGAAGGCCGCTGAGGCTGGAGCTTCGCAA
GCAGGTACATCTTTCTTGAAGGAGTTTGGTCTTGCAAGTTCCTCCTCATCAACCCCATCCAGGCCACCTCA
AGGAAGGTCTATGGATTGCTCAACACAATCCCAACGGCCCCAAGGAAGGTTCGTCGACAGCAGCTCAAT
ACAATCCCGATTACCTCAAGACAGGTTCATCGAGAGCAGCTCAACACAATGCCGGTTACCTCAAGACAG
GTTCATCGAGAGCAGCTCAACACAATCCCGGTTACCTCAAGACAGGTTCATCGACAGCAGTCCAACACA
ATCCCGGTTACCTCAAGACAGGTTCATCGACAGCAGTTCAACACAATTCCGGTTACCTCAAGACAGGTTC
ATCGACAACAGCTCAATACAATCCCGGGTACCTCAAGACAGGTTCATCGACACCAGCTCAACACAATCC
CGGTTACCTCAAGACAGGTTCATCGACAGCAGCTCAACACAATCCCGGTTACCTCAAGACAGGTTCATC
GACAGCAGCTCAACACAATCCCGGTTACCTCAAGACAGGTTCATCGATAACTCGATGCCATCCAAGCTTT
CTCAAGGGAGATTCATGGATACCTCACTGCCATCCCAGCAGCTGCTACCTCAAGGAAGGTTCTTTGACAA
CTCGCCGCCATCAAATCTGTCTCAAGGAAGGTTCTTCGAAAACTCGCAACCATCGAATCCACCTCAAGG
AAGGTTCTTCATCAACTCACCGCCATCTAATCTACCTCATGGAAGGTTCACTGATTACTCAACACCAGGAAT
GTTCATCGATAGCTCGACACTACCCAGGCTGCCTCAAGGAAGGTACATTGATAGCTTGCCACAGTCGAGG
CTACCACAAGGAAGGTACATGGATAACTCACCACCGGCCCAGCTTCCACAGAGAAGGTTGGCTGATAAC
AATCCAGAGCAGTGGCACCAAAGGAGCAATAATCATAACCAGCTAATGGATATGGAGCCTGGGCCGATG
GTATCTGGTAGCCTTGGCCTTGGCCTGGCCTATGAAGGTTCAAATCCAAGGTTGCCAGATTTGATGATGGG
GCAATCACCACTGTTCGGTCCCAAGCCTGCCACTCTGGACTTCCTTGGGCTTGGCATCGGAGGGACCAT
GGGCGGCGGCTCCACGGCGGCCAACGGTGGTGGCCTACCGGCATTGATGGTGGGTGGAGAGCTGGA

CATGGGGTCTGCCGCACAGGCGCCCTCTCCATGGGAGGAGGCACAGAGAAAGACCAACGGCCGCAC
GATCCTGTGA

>sdw3a_gDNA

ATGCCGCCCAATCCGACGGAGCCGGAGCAGCCGGAGGCGGCCGCGACGCCGGCGCCGCCCAAGA
AGAAGAGGAACCTCCCCGGGACGCCAGGCGAGAGCGCGTGCTTCGGATTTTGCGGGGTTGTTGGTAC
GGCTCGCGCGAGCTTTCTTAGTCTGACGGTGGTCGGTTTCCGTGTGCTTGTGCAGATCCGGACGCGGAG
GTGATCGCGCTGTCGCCGGGGACGCTCATGGCGACCAACCGGTTCGTGTGCGAGGTGTGCGGCAAGG
GCTTCCAGAGGGACCAGAACCTGCAGCTGCACCGCCGGGGGCACAACCTTCCGTGGCGGCTGCGG
CAGCGCGGCCCCGGGGCGGCGCCGCCGCGCCGGAGGGTCTACGTCTGCCCGGAGCCCGGCTGC
GTGCACCACTCCCCCGCCCGCGCGCTCGGGGACCTCACGGGTATCAAGAAGCACTTTTGCCGCAAG
CACGGCGAGAAGCGATGGGCCTGCCCACGCTGCGGCAAGCGCTACGCCGTCCAGGCCGACCTCAA
GGCCCATGCCAAGACCTGCGGCACCCGCGAGTATCGCTGTGACTGCGGCACGCTCTTCACCAAGTACT
ACTTCCAGCTCGGTTCCCCCCAATTTCGATGGCTCCTTTTTCCTTCCCTTGAGGGACAAATGTTTGTACTTT
TGTTTCATTCATCAGTCGGCGTGCTGGATTGATTCAGGAGAGACAGTTTCGTGACACATCGCGCTTTCTGTG
GCGCTCTCGTCGAGGAGACAGGCAGGGTGCTTGCTGTTCCGACGCCGCCTTCGCCTCGGCCACCTGA
TTTGGAGGAGGTTGAGGAGAATGTAGACAAGGACAAGGAAAAAGAAGAGGAGAATGTGGATAAGCACAA
GGAGAAGGAAGATGAGGAGGGCAAGGGGGGAGAAGATGAAAATGAGACTTCTGCCGTGGCCGAGGTG
GATGAGCCGCAGCACATTGAGGCAACAAGGGAGGAGCCACAGCCACAGCCACAGCGGACTCCGTCG
CCGCCATCTCCAATGCCACAGGAGCAGCACCCAATGGTGGCAATTGTGCCAAATTTGGATGGTATGCTT
CTTTTGCATTTTGCAATGTGTTTGTCTTACTACCTATTCATAGACCATGTTAGATTTATAGCAAACTCATTCTCC
CTAATCCATCTTTATTTCTTATCACTAGCAAAAAAAGGAATGCATCAGTCTTGTTTCTTTCCCAATTTAGCTAG
CCTTATGATCATTCAGTCAACAATGATGAGTGACCTGTTCTTTTGTACCATGCCTATATGTTACAGAGCCAGT
GGTGGTTGTGGAGCCAATTGTGGATATCAAGCAAGAGGAGGAAGATAAGCGAGATGAAGATGTTTGCTTC
CAGGAAGCAGATAATTACGGCGATGCTGAACTAGAAGACTCCAACTTGCCAGATAATGATACCCCGATGC
CTCCTTGTTTCCTCCCATCGCCCTCGGATGCCATTGGTACAGATGGCAGCAGCACCAGTTGTGGCACAG
TCAGCAGCGCATCCAATTCCATCGTGCCAGCAACGACGACTAGCACATTTGCGGGGCTGTTTGCATCAG
CCACAAAAAGCACCACTCCCCAGAGCAGATCGCTGCGTGATCTTATCGGTGTTGATCCCACCTTCCTTTG
CCTTGCAATCGGCACACCATCCTCTCTGTTCCCGCAGACAGATGCAAGCAACCCCAGCACCTTTGCTCC
ACCTCCAGCACCACACATCTCCGCAACTGCGCTCCTGCAGAAGGCCGCTGAGGCTGGAGCTTCGCAA
GCAGGTACATCTTTCTTGAAGGAGTTTGGTCTTGCAAGTTCCTCCTCATCAACCCCATCCAGGCCACCTCA
AGGAAGGTCTATGGATTGCTCAACACAATCCCAACGGCCCCAAGGAAGGTTCGTCGACAGCAGCTCAAT
ACAATCCCGATTACCTCAAGACAGGTTCATCGAGAGCAGCTCAACACAATGCCGGTTACCTCAAGACAG
GTTCATCGAGAGCAGCTCAACACAATCCCGGTTACCTCAAGACAGGTTCATCGACAGCAGTCCAACACA
ATCCCGGTTACCTCAAGACAGGTTCATCGACAGCAGTTCAACACAATTCCGGTTACCTCAAGACAGGTTC
ATCGACAACAGCTCAATACAATCCCGGGTACCTCAAGACAGGTTCATCGACACCAGCTCAACACAATCC
CGGTTACCTCAAGACAGGTTCATCGACAGCAGCTCAACACAATCCCGGTTACCTCAAGACAGGTTCATC
GACAGCAGCTCAACACAATCCCGGTTACCTCAAGACAGGTTCATCGATAACTCGATGCCATCCAAGCTTT
CTCAAGGGAGATTCATGGATACCTCACTGCCATCCCAGCAGCTGCTACCTCAAGGAAGGTTCTTTGACAA
CTCGCCGCCATCAAATCTGTCTCAAGGAAGGTTCTTCGAAAACTCGCAACCATCGAATCCACCTCAAGG
AAGGTTCTTCATCAACTCACCGCCATCTAATCTACCTCATGGAAGGTTCACTGATTACTCAACACCAGGAAT
GTTCATCGATAGCTCGACACTACCCAGGCTGCCTCAAGGAAGGTACATTGATAGCTTGCCACAGTCGAGG
CTACCACAAGGAAGGTACATGGATAACTCACCACCGGCCCAGCTTCCACAGAGAAGGTTGGCTGATAAC
AATCCAGAGCAGTGGCACCAAAGGAGCAATAATCATAACCAGCTAATGGATATGGAGCCTGGGCCGATG

GTATCTGGTAGCCTTGGCCTTGGCCTGGCCTATGAAGGTTCAAATCCAAGGTTGCCAGATTTGATGATGGG
GCAATCACCACTGTTCGGTCCCAAGCCTGCCACTCTGGACTTCCTTGGGCTTGGCATCGGAGGGACCAT
GGGCGGCGGCTCCACGGCGGCCAACGGTGGTGGCCTACCGGCATTGATGGTGGGTGGAGAGCTGGA
CATGGGGTCTGCCGCACAGGCGCCCTCTCCATGGGAGGAGGCACAGAGAAAGACCAACGGCCGCAC
GATCCTGTGA

>sdw3b_gDNA

ATGCCGCCCAATCCGACGGAGCCGGAGCAGCCGGAGGCGGCCGCGACGCCGGCGCCGCCCAAGA
AGAAGAGGAACCTCCCCGGGACGCCAGGCGAGAGCGCGTGCTTCGGATTTTGCGGGGTTGTTGGTAC
GGCTCGCGCGAGCTTTCTTAGTCTGACGGTGGTCGGTTTCCGTGTGCTTGTGCAGATCCGGACGCGGAG
GTGATCGCGCTGTTGCCGGGGACGCTCATGGCGACCAACCGGTTCGTGTGCGAGGTGTGCGGCAAGG
GCTTCCAGAGGGACCAGAACCTGCAGCTGCACCGCCGGGGGCACAACCTTCCGTGGCGGCTGCGG
CAGCGCGGCCCCGGGGCGGCGCCGCCGCGCCGGAGGGTCTACGTCTGCCCGGAGCCCGGCTGC
GTGCACCACTCCCCCGCCCGCGCGCTCGGGGACCTCACGGGTATCAAGAAGCACTTTTGCCGCAAG
CACGGCGAGAAGCGATGGGCCTGCCCACGCTGCGGCAAGCGCTACGCCGTCCAGGCCGACCTCAA
GGCCCATGCCAAGACCTGCGGCACCCGCGAGTATCGCTGTGACTGCGGCACGCTCTTCACCAGGTAC
TACTTCCAGCTCGGTTCCCCCCAATTTCGATGGCTCCTTTTTCCTTCCCTTGAGGGACAAATGTTTGTACTT
TTGTTTCATTCATCAGTCGGCGTGCTGGATTGATTCAGGAGAGACAGTTTCGTGACACATCGCGCTTTCTGT
GGCGCTCTCGTCGAGGAGACAGGCAGGGTGCTTGCTGTTCCGACGCCGCCTTCGCCTCGGCCACCTG
ATTTGGAGGAGGTTGAGGAGAATGTAGACAAGGACAAGGAAAAAGAAGAGGAGAATGTGGATAAGCACAA
GGAGAAGGAAGATGAGGAGGGCAAGGGGGGAGAAGATGAAAATGAGACTTCTGCCGTGGCCGAGGTG
GATGAGCCGCAGCACATTGAGGCAACAAGGGAGGAGCCACAGCCACAGCCACAGCGGACTCCGTCG
CCGCCATCTCCAATGCCACAGGAGCAGCACCCAATGGTGGCAATTGTGCCAAATTTGGATGGTATGCTT
CTTTTGCATTTTGCAATGTGTTTGTCTTACTACCTATTCATAGACCATGTTAGATTTATAGCAAACTCATTCTCC
CTAATCCATCTTTATTTCTTATCACTAGCAAAAAAAGGAATGCATCAGTCTTGTTTCTTTCCCAATTTAGCTAG
CCTTATGATCATTCAGTCAACAATGATGAGTGACCTGTTCTTTTGTACCATGCCTATATGTTACAGAGCCAGT
GGTGGTTGTGGAGCCAATTGTGGATATCAAGCAAGAGGAGGAAGATAAGCGAGATGAAGATGTTTGCTTC
CAGGAAGCAGATAATTACGGCGATGCTGAACTAGAAGACTCCAACTTGCCAGATAATGATACCCCGATGC
CTCCTTGTTTCCTCCCATCGCCCTCGGATGCCATTGGTACAGATGGCAGCAGCACCAGTTGTGGCACAG
TCAGCAGCGCATCCAATTCCATCGTGCCAGCAACGACGACTAGCACATTTGCGGGGCTGTTTGCATCAG
CCACAAAAAGCACCACTCCCCAGAGCAGATCGCTGCGTGATCTTATCGGTGTTGATCCCACCTTCCTTTG
CCTTGCAATCGGCACACCATCCTCTCTGTTCCCGCAGACAGATGCAAGCAACCCCAGCACCTTTGCTCC
ACCTCCAGCACCACACATCTCCGCAACTGCGCTCCTGCAGAAGGCCGCTGAGGCTGGAGCTTCGCAA
GCAGGTACATCTTTCTTGAAGGAGTTTGGTCTTGCAAGTTCCTCCTCATCAACCCCATCCAGGCCACCTCA
AGGAAGGTCTATGGATTGCTCAACACAATCCCAACGGCCCCAAGGAAGGTTCGTCGACAGCAGCTCAAT
ACAATCCCGATTACCTCAAGACAGGTTCATCGAGAGCAGCTCAACACAATGCCGGTTACCTCAAGACAG
GTTCATCGAGAGCAGCTCAACACAATCCCGGTTACCTCAAGACAGGTTCATCGACAGCAGTCCAACACA
ATCCCGGTTACCTCAAGACAGGTTCATCGACAGCAGTTCAACACAATTCCGGTTACCTCAAGACAGGTTC
ATCGACAACAGCTCAATACAATCCCGGGTACCTCAAGACAGGTTCATCGACACCAGCTCAACACAATCC
CGGTTACCTCAAGACAGGTTCATCGACAGCAGCTCAACACAATCCCGGTTACCTCAAGACAGGTTCATC
GACAGCAGCTCAACACAATCCCGGTTACCTCAAGACAGGTTCATCGATAACTCGATGCCATCCAAGCTTT
CTCAAGGGAGATTCATGGATACCTCACTGCCATCCCAGCAGCTGCTACCTCAAGGAAGGTTCTTTGACAA
CTCGCCGCCATCAAATCTGTCTCAAGGAAGGTTCTTCGAAAACTCGCAACCATCGAATCCACCTCAAGG
AAGGTTCTTCATCAACTCACCGCCATCTAATCTACCTCATGGAAGGTTCACTGATTACTCAACACCAGGAAT

GTTCATCGATAGCTCGACACTACCCAGGCTGCCTCAAGGAAGGTACATTGATAGCTTGCCACAGTCGAGG
CTACCACAAGGAAGGTACATGGATAACTCACCACCGGCCCAGCTTCCACAGAGAAGGTTGGCTGATAAC
AATCCAGAGCAGTGGCACCAAAGGAGCAATAATCATAACCAGCTAATGGATATGGAGCCTGGGCCGATG
GTATCTGGTAGCCTTGGCCTTGGCCTGGCCTATGAAGGTTCAAATCCAAGGTTGCCAGATTTGATGATGGG
GCAATCACCACTGTTCGGTCCCAAGCCTGCCACTCTGGACTTCCTTGGGCTTGGCATCGGAGGGACCAT
GGGCGGCGGCTCCACGGCGGCCAACGGTGGTGGCCTACCGGCATTGATGGTGGGTGGAGAGCTGGA
CATGGGGTCTGCCGCACAGGCGCCCTCTCCATGGGAGGAGGCACAGAGAAAGACCAACGGCCGCAC
GATCCTGTGA

>sdw3c_gDNA

ATGCCGCCCAATCCGACGGAGCCGGAGCAGCCGGAGGCGGCCGCGACGCCGGCGCCGCCCAAGA
AGAAGAGGAACCTCCCCGGGACGCCAGGCGAGAGCGCGTGCTTCGGATTTTGCGGGGTTGTTGGTAC
GGCTCGCGCGAGCTTTCTTAGTCTGACGGTGGTCGGTTTCCGTGTGCTTGTGCAGATCCGGACGCGGAG
GTGATCGCGCTGTCGCCGGGGACGCTCATGGCGACCAACCGGTTCGTGTGCGAGGTGTGCGGCAAGG
GCTTCCAGAAGGACCAGAACCTGCAGCTGCACCGCCGGGGGCACAACCTTCCGTGGCGGCTGCGGC
AGCGCGGCCCCGGGGCGGCGCCGCCGCGCCGGAGGGTCTACGTCTGCCCGGAGCCCGGCTGCG
TGCACCACTCCCCCGCCCGCGCGCTCGGGGACCTCACGGGTATCAAGAAGCACTTTTGCCGCAAGCA
CGGCGAGAAGCGATGGGCCTGCCCACGCTGCGGCAAGCGCTACGCCGTCCAGGCCGACCTCAAGG
CCCATGCCAAGACCTGCGGCACCCGCGAGTATCGCTGTGACTGCGGCACGCTCTTCACCAGGTACTAC
TTCCAGCTCGGTTCCCCCCAATTTCGATGGCTCCTTTTTCCTTCCCTTGAGGGACAAATGTTTGTACTTTTG
TTTCATTCATCAGTCGGCGTGCTGGATTGATTCAGGAGAGACAGTTTCGTGACACATCGCGCTTTCTGTGG
CGCTCTCGTCGAGGAGACAGGCAGGGTGCTTGCTGTTCCGACGCCGCCTTCGCCTCGGCCACCTGAT
TTGGAGGAGGTTGAGGAGAATGTAGACAAGGACAAGGAAAAAGAAGAGGAGAATGTGGATAAGCACAAG
GAGAAGGAAGATGAGGAGGGCAAGGGGGGAGAAGATGAAAATGAGACTTCTGCCGTGGCCGAGGTGG
ATGAGCCGCAGCACATTGAGGCAACAAGGGAGGAGCCACAGCCACAGCCACAGCGGACTCCGTCGC
CGCCATCTCCAATGCCACAGGAGCAGCACCCAATGGTGGCAATTGTGCCAAATTTGGATGGTATGCTTCT
TTTGCATTTTGCAATGTGTTTGTCTTACTACCTATTCATAGACCATGTTAGATTTATAGCAAACTCATTCTCCCTA
ATCCATCTTTATTTCTTATCACTAGCAAAAAAAGGAATGCATCAGTCTTGTTTCTTTCCCAATTTAGCTAGCCTT
ATGATCATTCAGTCAACAATGATGAGTGACCTGTTCTTTTGTACCATGCCTATATGTTACAGAGCCAGTGGTG
GTTGTGGAGCCAATTGTGGATATCAAGCAAGAGGAGGAAGATAAGCGAGATGAAGATGTTTGCTTCCAGG
AAGCAGATAATTACGGCGATGCTGAACTAGAAGACTCCAACTTGCCAGATAATGATACCCCGATGCCTCC
TTGTTTCCTCCCATCGCCCTCGGATGCCATTGGTACAGATGGCAGCAGCACCAGTTGTGGCACAGTCAG
CAGCGCATCCAATTCCATCGTGCCAGCAACGACGACTAGCACATTTGCGGGGCTGTTTGCATCAGCCAC
AAAAAGCACCACTCCCCAGAGCAGATCGCTGCGTGATCTTATCGGTGTTGATCCCACCTTCCTTTGCCTT
GCAATCGGCACACCATCCTCTCTGTTCCCGCAGACAGATGCAAGCAACCCCAGCACCTTTGCTCCACC
TCCAGCACCACACATCTCCGCAACTGCGCTCCTGCAGAAGGCCGCTGAGGCTGGAGCTTCGCAAGCA
GGTACATCTTTCTTGAAGGAGTTTGGTCTTGCAAGTTCCTCCTCATCAACCCCATCCAGGCCACCTCAAG
GAAGGTCTATGGATTGCTCAACACAATCCCAACGGCCCCAAGGAAGGTTCGTCGACAGCAGCTCAATAC
AATCCCGATTACCTCAAGACAGGTTCATCGAGAGCAGCTCAACACAATGCCGGTTACCTCAAGACAGGTT
CATCGAGAGCAGCTCAACACAATCCCGGTTACCTCAAGACAGGTTCATCGACAGCAGTCCAACACAATC
CCGGTTACCTCAAGACAGGTTCATCGACAGCAGTTCAACACAATTCCGGTTACCTCAAGACAGGTTCATC
GACAACAGCTCAATACAATCCCGGGTACCTCAAGACAGGTTCATCGACACCAGCTCAACACAATCCCGG
TTACCTCAAGACAGGTTCATCGACAGCAGCTCAACACAATCCCGGTTACCTCAAGACAGGTTCATCGACA
GCAGCTCAACACAATCCCGGTTACCTCAAGACAGGTTCATCGATAACTCGATGCCATCCAAGCTTTCTCA

AGGGAGATTCATGGATACCTCACTGCCATCCCAGCAGCTGCTACCTCAAGGAAGGTTCTTTGACAACTCG
CCGCCATCAAATCTGTCTCAAGGAAGGTTCTTCGAAAACTCGCAACCATCGAATCCACCTCAAGGAAGGT
TCTTCATCAACTCACCGCCATCTAATCTACCTCATGGAAGGTTCACTGATTACTCAACACCAGGAATGTTCA
TCGATAGCTCGACACTACCCAGGCTGCCTCAAGGAAGGTACATTGATAGCTTGCCACAGTCGAGGCTAC
CACAAGGAAGGTACATGGATAACTCACCACCGGCCCAGCTTCCACAGAGAAGGTTGGCTGATAACAATC
CAGAGCAGTGGCACCAAAGGAGCAATAATCATAACCAGCTAATGGATATGGAGCCTGGGCCGATGGTAT
CTGGTAGCCTTGGCCTTGGCCTGGCCTATGAAGGTTCAAATCCAAGGTTGCCAGATTTGATGATGGGGCA
ATCACCACTGTTCGGTCCCAAGCCTGCCACTCTGGACTTCCTTGGGCTTGGCATCGGAGGGACCATGG
GCGGCGGCTCCACGGCGGCCAACGGTGGTGGCCTACCGGCATTGATGGTGGGTGGAGAGCTGGACA
TGGGGTCTGCCGCACAGGCGCCCTCTCCATGGGAGGAGGCACAGAGAAAGACCAACGGCCGCACGA
TCCTGTGA

>sdw3d_gDNA

ATGCCGCCCAATCCGACGGAGCCGGAGCAGCCGGAGGCGGCCGCGACGCCGGCGCCGCCCAAGA
AGAAGAGGAACCTCCCCGGGACGCCAGGCGAGAGCGCGTGCTTCGGATTTTGCGGGGTTGTTGGTAC
GGCTCGCGCGAGCTTTCTTAGTCTGACGGTGGTCGGTTTCCGTGTGCTTGTGCAGATCCGGACGCGGAG
GTGATCGCGCTGTCGCCGGGGACGCTCATGGCGACCAACCGGTTCGTGTGCGAGGTGTGCGGCAAGG
GCTTCCAGAGGGACCAGAACCTGCAGCTGCACCGCCGGGGGCACAACCTTCCGTGGCGGCTGCGG
CAGCGCGGCCCCGGGGCGGCGCCGCCGCGCCGGAGGGTCTACGTCTGCCCGGAGCCCGGCTGC
GTGCACCACTCCCTCGCCCGCGCGCTCGGGGACCTCACGGGTATCAAGAAGCACTTTTGCCGCAAGC
ACGGCGAGAAGCGATGGGCCTGCCCACGCTGCGGCAAGCGCTACGCCGTCCAGGCCGACCTCAAG
GCCCATGCCAAGACCTGCGGCACCCGCGAGTATCGCTGTGACTGCGGCACGCTCTTCACCAGGTACTA
CTTCCAGCTCGGTTCCCCCCAATTTCGATGGCTCCTTTTTCCTTCCCTTGAGGGACAAATGTTTGTACTTTT
GTTTCATTCATCAGTCGGCGTGCTGGATTGATTCAGGAGAGACAGTTTCGTGACACATCGCGCTTTCTGTG
GCGCTCTCGTCGAGGAGACAGGCAGGGTGCTTGCTGTTCCGACGCCGCCTTCGCCTCGGCCACCTGA
TTTGGAGGAGGTTGAGGAGAATGTAGACAAGGACAAGGAAAAAGAAGAGGAGAATGTGGATAAGCACAA
GGAGAAGGAAGATGAGGAGGGCAAGGGGGGAGAAGATGAAAATGAGACTTCTGCCGTGGCCGAGGTG
GATGAGCCGCAGCACATTGAGGCAACAAGGGAGGAGCCACAGCCACAGCCACAGCGGACTCCGTCG
CCGCCATCTCCAATGCCACAGGAGCAGCACCCAATGGTGGCAATTGTGCCAAATTTGGATGGTATGCTT
CTTTTGCATTTTGCAATGTGTTTGTCTTACTACCTATTCATAGACCATGTTAGATTTATAGCAAACTCATTCTCC
CTAATCCATCTTTATTTCTTATCACTAGCAAAAAAAGGAATGCATCAGTCTTGTTTCTTTCCCAATTTAGCTAG
CCTTATGATCATTCAGTCAACAATGATGAGTGACCTGTTCTTTTGTACCATGCCTATATGTTACAGAGCCAGT
GGTGGTTGTGGAGCCAATTGTGGATATCAAGCAAGAGGAGGAAGATAAGCGAGATGAAGATGTTTGCTTC
CAGGAAGCAGATAATTACGGCGATGCTGAACTAGAAGACTCCAACTTGCCAGATAATGATACCCCGATGC
CTCCTTGTTTCCTCCCATCGCCCTCGGATGCCATTGGTACAGATGGCAGCAGCACCAGTTGTGGCACAG
TCAGCAGCGCATCCAATTCCATCGTGCCAGCAACGACGACTAGCACATTTGCGGGGCTGTTTGCATCAG
CCACAAAAAGCACCACTCCCCAGAGCAGATCGCTGCGTGATCTTATCGGTGTTGATCCCACCTTCCTTTG
CCTTGCAATCGGCACACCATCCTCTCTGTTCCCGCAGACAGATGCAAGCAACCCCAGCACCTTTGCTCC
ACCTCCAGCACCACACATCTCCGCAACTGCGCTCCTGCAGAAGGCCGCTGAGGCTGGAGCTTCGCAA
GCAGGTACATCTTTCTTGAAGGAGTTTGGTCTTGCAAGTTCCTCCTCATCAACCCCATCCAGGCCACCTCA
AGGAAGGTCTATGGATTGCTCAACACAATCCCAACGGCCCCAAGGAAGGTTCGTCGACAGCAGCTCAAT
ACAATCCCGATTACCTCAAGACAGGTTCATCGAGAGCAGCTCAACACAATGCCGGTTACCTCAAGACAG
GTTCATCGAGAGCAGCTCAACACAATCCCGGTTACCTCAAGACAGGTTCATCGACAGCAGTCCAACACA
ATCCCGGTTACCTCAAGACAGGTTCATCGACAGCAGTTCAACACAATTCCGGTTACCTCAAGACAGGTTC

ATCGACAACAGCTCAATACAATCCCGGGTACCTCAAGACAGGTTCATCGACACCAGCTCAACACAATCC
CGGTTACCTCAAGACAGGTTCATCGACAGCAGCTCAACACAATCCCGGTTACCTCAAGACAGGTTCATC
GACAGCAGCTCAACACAATCCCGGTTACCTCAAGACAGGTTCATCGATAACTCGATGCCATCCAAGCTTT
CTCAAGGGAGATTCATGGATACCTCACTGCCATCCCAGCAGCTGCTACCTCAAGGAAGGTTCTTTGACAA
CTCGCCGCCATCAAATCTGTCTCAAGGAAGGTTCTTCGAAAACTCGCAACCATCGAATCCACCTCAAGG
AAGGTTCTTCATCAACTCACCGCCATCTAATCTACCTCATGGAAGGTTCACTGATTACTCAACACCAGGAAT
GTTCATCGATAGCTCGACACTACCCAGGCTGCCTCAAGGAAGGTACATTGATAGCTTGCCACAGTCGAGG
CTACCACAAGGAAGGTACATGGATAACTCACCACCGGCCCAGCTTCCACAGAGAAGGTTGGCTGATAAC
AATCCAGAGCAGTGGCACCAAAGGAGCAATAATCATAACCAGCTAATGGATATGGAGCCTGGGCCGATG
GTATCTGGTAGCCTTGGCCTTGGCCTGGCCTATGAAGGTTCAAATCCAAGGTTGCCAGATTTGATGATGGG
GCAATCACCACTGTTCGGTCCCAAGCCTGCCACTCTGGACTTCCTTGGGCTTGGCATCGGAGGGACCAT
GGGCGGCGGCTCCACGGCGGCCAACGGTGGTGGCCTACCGGCATTGATGGTGGGTGGAGAGCTGGA
CATGGGGTCTGCCGCACAGGCGCCCTCTCCATGGGAGGAGGCACAGAGAAAGACCAACGGCCGCAC
GATCCTGTGA

>sdw3e_gDNA

ATGCCGCCCAATCCGACGGAGCCGGAGCAGCCGGAGGCGGCCGCGACGCCGGCGCCGCCCAAGA
AGAAGAGGAACCTCCCCGGGACGCCAGGCGAGAGCGCGTGCTTCGGATTTTGCGGGGTTGTTGGTAC
GGCTCGCGCGAGCTTTCTTAGTCTGACGGTGGTCGGTTTCCGTGTGCTTGTGCAGATCCGGACGCGGAG
GTGATCGCGCTGTCGCCGGGGACGCTCATGGCGACCAACCGGTTCGTGTGCGAGGTGTGCGGCAAGG
GCTTCCAGAGGGACCAGAACCTGCAGCTGCACCGCCGGGGGCACAACCTTCCGTGGCGGCTGCGG
CAGCGCGGCCCCGGGGCGGCGCCGCCGCGCCGGAGGGTCTACGTCTGCCCGGAGCCCGGCTGC
GTGCACCACTCCCCCGCCCGCGCGCTCGAGGACCTCACGGGTATCAAGAAGCACTTTTGCCGCAAGC
ACGGCGAGAAGCGATGGGCCTGCCCACGCTGCGGCAAGCGCTACGCCGTCCAGGCCGACCTCAAG
GCCCATGCCAAGACCTGCGGCACCCGCGAGTATCGCTGTGACTGCGGCACGCTCTTCACCAGGTACTA
CTTCCAGCTCGGTTCCCCCCAATTTCGATGGCTCCTTTTTCCTTCCCTTGAGGGACAAATGTTTGTACTTTT
GTTTCATTCATCAGTCGGCGTGCTGGATTGATTCAGGAGAGACAGTTTCGTGACACATCGCGCTTTCTGTG
GCGCTCTCGTCGAGGAGACAGGCAGGGTGCTTGCTGTTCCGACGCCGCCTTCGCCTCGGCCACCTGA
TTTGGAGGAGGTTGAGGAGAATGTAGACAAGGACAAGGAAAAAGAAGAGGAGAATGTGGATAAGCACAA
GGAGAAGGAAGATGAGGAGGGCAAGGGGGGAGAAGATGAAAATGAGACTTCTGCCGTGGCCGAGGTG
GATGAGCCGCAGCACATTGAGGCAACAAGGGAGGAGCCACAGCCACAGCCACAGCGGACTCCGTCG
CCGCCATCTCCAATGCCACAGGAGCAGCACCCAATGGTGGCAATTGTGCCAAATTTGGATGGTATGCTT
CTTTTGCATTTTGCAATGTGTTTGTCTTACTACCTATTCATAGACCATGTTAGATTTATAGCAAACTCATTCTCC
CTAATCCATCTTTATTTCTTATCACTAGCAAAAAAAGGAATGCATCAGTCTTGTTTCTTTCCCAATTTAGCTAG
CCTTATGATCATTCAGTCAACAATGATGAGTGACCTGTTCTTTTGTACCATGCCTATATGTTACAGAGCCAGT
GGTGGTTGTGGAGCCAATTGTGGATATCAAGCAAGAGGAGGAAGATAAGCGAGATGAAGATGTTTGCTTC
CAGGAAGCAGATAATTACGGCGATGCTGAACTAGAAGACTCCAACTTGCCAGATAATGATACCCCGATGC
CTCCTTGTTTCCTCCCATCGCCCTCGGATGCCATTGGTACAGATGGCAGCAGCACCAGTTGTGGCACAG
TCAGCAGCGCATCCAATTCCATCGTGCCAGCAACGACGACTAGCACATTTGCGGGGCTGTTTGCATCAG
CCACAAAAAGCACCACTCCCCAGAGCAGATCGCTGCGTGATCTTATCGGTGTTGATCCCACCTTCCTTTG
CCTTGCAATCGGCACACCATCCTCTCTGTTCCCGCAGACAGATGCAAGCAACCCCAGCACCTTTGCTCC
ACCTCCAGCACCACACATCTCCGCAACTGCGCTCCTGCAGAAGGCCGCTGAGGCTGGAGCTTCGCAA
GCAGGTACATCTTTCTTGAAGGAGTTTGGTCTTGCAAGTTCCTCCTCATCAACCCCATCCAGGCCACCTCA
AGGAAGGTCTATGGATTGCTCAACACAATCCCAACGGCCCCAAGGAAGGTTCGTCGACAGCAGCTCAAT

ACAATCCCGATTACCTCAAGACAGGTTCATCGAGAGCAGCTCAACACAATGCCGGTTACCTCAAGACAG
GTTCATCGAGAGCAGCTCAACACAATCCCGGTTACCTCAAGACAGGTTCATCGACAGCAGTCCAACACA
ATCCCGGTTACCTCAAGACAGGTTCATCGACAGCAGTTCAACACAATTCCGGTTACCTCAAGACAGGTTC
ATCGACAACAGCTCAATACAATCCCGGGTACCTCAAGACAGGTTCATCGACACCAGCTCAACACAATCC
CGGTTACCTCAAGACAGGTTCATCGACAGCAGCTCAACACAATCCCGGTTACCTCAAGACAGGTTCATC
GACAGCAGCTCAACACAATCCCGGTTACCTCAAGACAGGTTCATCGATAACTCGATGCCATCCAAGCTTT
CTCAAGGGAGATTCATGGATACCTCACTGCCATCCCAGCAGCTGCTACCTCAAGGAAGGTTCTTTGACAA
CTCGCCGCCATCAAATCTGTCTCAAGGAAGGTTCTTCGAAAACTCGCAACCATCGAATCCACCTCAAGG
AAGGTTCTTCATCAACTCACCGCCATCTAATCTACCTCATGGAAGGTTCACTGATTACTCAACACCAGGAAT
GTTCATCGATAGCTCGACACTACCCAGGCTGCCTCAAGGAAGGTACATTGATAGCTTGCCACAGTCGAGG
CTACCACAAGGAAGGTACATGGATAACTCACCACCGGCCCAGCTTCCACAGAGAAGGTTGGCTGATAAC
AATCCAGAGCAGTGGCACCAAAGGAGCAATAATCATAACCAGCTAATGGATATGGAGCCTGGGCCGATG
GTATCTGGTAGCCTTGGCCTTGGCCTGGCCTATGAAGGTTCAAATCCAAGGTTGCCAGATTTGATGATGGG
GCAATCACCACTGTTCGGTCCCAAGCCTGCCACTCTGGACTTCCTTGGGCTTGGCATCGGAGGGACCAT
GGGCGGCGGCTCCACGGCGGCCAACGGTGGTGGCCTACCGGCATTGATGGTGGGTGGAGAGCTGGA
CATGGGGTCTGCCGCACAGGCGCCCTCTCCATGGGAGGAGGCACAGAGAAAGACCAACGGCCGCAC
GATCCTGTGA

>sdw3f_gDNA

ATGCCGCCCAATCCGACGGAGCCGGAGCAGCCGGAGGCGGCCGCGACGCCGGCGCCGCCCAAGA
AGAAGAGGAACCTCCCCGGGACGCCAGGCGAGAGCGCGTGCTTCGGATTTTGCGGGGTTGTTGGTAC
GGCTCGCGCGAGCTTTCTTAGTCTGACGGTGGTCGGTTTCCGTGTGCTTGTGCAGATCCGGACGCGGAG
GTGATCGCGCTGTCGCCGGGGACGCTCATGGCGACCAACCGGTTCGTGTGCGAGGTGTGCGGCAAGG
GCTTCCAGAGGGACCAGAACCTGCAGCTGCACCGCCGGGGGCACAACCTTCCGTGGCGGCTGCGG
CAGCGCGGCCCCGGGGCGGCGCCGCCGCGCCGGAGGGTCTACGTCTGCCCGGAGCCCGGCTGC
GTGCACCACTCCCCCGCCCGCGCGCTCGGGGACCTCACGGGTATCAAGAAGCACTTTTGCCGCAAG
CACGGCGAGAAGCGATGGGCCTGCCCACGCTGCGGCAAGCGCTACGCCGTCCAGGCCGACCTCAA
GGCCCATGCCAAGACCTGCGGCACCCGCGAGTATCGCTGTGACTGCGGCACGCTCTTCACCAGGTAC
TACTTCCAGCTCGGTTCCCCCCAATTTCGATGGCTCCTTTTTCCTTCCCTTGAGGGACAAATGTTTGTACTT
TTGTTTCATTCATCAGTCGGCGTGCTGGATTGATTCAGGAGAGACAGTTTCGTGACACATCGCGCTTTCTGT
GGCGCTCTCGTCGAGGAGACAGGCAGGGTGCTTGCTGTTCCGACGCCGCCTTCGCCTCGGCCACCTG
ATTTGGAGGAGGTTGAGGAGAATGTAGACAAGGACAAGTAAAAAGAAGAGGAGAATGTGGATAAGCACAA
GGAGAAGGAAGATGAGGAGGGCAAGGGGGGAGAAGATGAAAATGAGACTTCTGCCGTGGCCGAGGTG
GATGAGCCGCAGCACATTGAGGCAACAAGGGAGGAGCCACAGCCACAGCCACAGCGGACTCCGTCG
CCGCCATCTCCAATGCCACAGGAGCAGCACCCAATGGTGGCAATTGTGCCAAATTTGGATGGTATGCTT
CTTTTGCATTTTGCAATGTGTTTGTCTTACTACCTATTCATAGACCATGTTAGATTTATAGCAAACTCATTCTCC
CTAATCCATCTTTATTTCTTATCACTAGCAAAAAAAGGAATGCATCAGTCTTGTTTCTTTCCCAATTTAGCTAG
CCTTATGATCATTCAGTCAACAATGATGAGTGACCTGTTCTTTTGTACCATGCCTATATGTTACAGAGCCAGT
GGTGGTTGTGGAGCCAATTGTGGATATCAAGCAAGAGGAGGAAGATAAGCGAGATGAAGATGTTTGCTTC
CAGGAAGCAGATAATTACGGCGATGCTGAACTAGAAGACTCCAACTTGCCAGATAATGATACCCCGATGC
CTCCTTGTTTCCTCCCATCGCCCTCGGATGCCATTGGTACAGATGGCAGCAGCACCAGTTGTGGCACAG
TCAGCAGCGCATCCAATTCCATCGTGCCAGCAACGACGACTAGCACATTTGCGGGGCTGTTTGCATCAG
CCACAAAAAGCACCACTCCCCAGAGCAGATCGCTGCGTGATCTTATCGGTGTTGATCCCACCTTCCTTTG
CCTTGCAATCGGCACACCATCCTCTCTGTTCCCGCAGACAGATGCAAGCAACCCCAGCACCTTTGCTCC

ACCTCCAGCACCACACATCTCCGCAACTGCGCTCCTGCAGAAGGCCGCTGAGGCTGGAGCTTCGCAA
GCAGGTACATCTTTCTTGAAGGAGTTTGGTCTTGCAAGTTCCTCCTCATCAACCCCATCCAGGCCACCTCA
AGGAAGGTCTATGGATTGCTCAACACAATCCCAACGGCCCCAAGGAAGGTTCGTCGACAGCAGCTCAAT
ACAATCCCGATTACCTCAAGACAGGTTCATCGAGAGCAGCTCAACACAATGCCGGTTACCTCAAGACAG
GTTCATCGAGAGCAGCTCAACACAATCCCGGTTACCTCAAGACAGGTTCATCGACAGCAGTCCAACACA
ATCCCGGTTACCTCAAGACAGGTTCATCGACAGCAGTTCAACACAATTCCGGTTACCTCAAGACAGGTTC
ATCGACAACAGCTCAATACAATCCCGGGTACCTCAAGACAGGTTCATCGACACCAGCTCAACACAATCC
CGGTTACCTCAAGACAGGTTCATCGACAGCAGCTCAACACAATCCCGGTTACCTCAAGACAGGTTCATC
GACAGCAGCTCAACACAATCCCGGTTACCTCAAGACAGGTTCATCGATAACTCGATGCCATCCAAGCTTT
CTCAAGGGAGATTCATGGATACCTCACTGCCATCCCAGCAGCTGCTACCTCAAGGAAGGTTCTTTGACAA
CTCGCCGCCATCAAATCTGTCTCAAGGAAGGTTCTTCGAAAACTCGCAACCATCGAATCCACCTCAAGG
AAGGTTCTTCATCAACTCACCGCCATCTAATCTACCTCATGGAAGGTTCACTGATTACTCAACACCAGGAAT
GTTCATCGATAGCTCGACACTACCCAGGCTGCCTCAAGGAAGGTACATTGATAGCTTGCCACAGTCGAGG
CTACCACAAGGAAGGTACATGGATAACTCACCACCGGCCCAGCTTCCACAGAGAAGGTTGGCTGATAAC
AATCCAGAGCAGTGGCACCAAAGGAGCAATAATCATAACCAGCTAATGGATATGGAGCCTGGGCCGATG
GTATCTGGTAGCCTTGGCCTTGGCCTGGCCTATGAAGGTTCAAATCCAAGGTTGCCAGATTTGATGATGGG
GCAATCACCACTGTTCGGTCCCAAGCCTGCCACTCTGGACTTCCTTGGGCTTGGCATCGGAGGGACCAT
GGGCGGCGGCTCCACGGCGGCCAACGGTGGTGGCCTACCGGCATTGATGGTGGGTGGAGAGCTGGA
CATGGGGTCTGCCGCACAGGCGCCCTCTCCATGGGAGGAGGCACAGAGAAAGACCAACGGCCGCAC
GATCCTGTGA

>sdw3g_gDNA

ATGCCGCCCAATCCGACGGAGCCGGAGCAGCCGGAGGCGGCCGCGACGCCGGCGCCGCCCAAGA
AGAAGAGGAACCTCCCCGGGACGCCAGGCGAGAGCGCGTGCTTCGGATTTTGCGGGGTTGTTGGTAC
GGCTCGCGCGAGCTTTCTTAGTCTGACGGTGGTCGGTTTCCGTGTGCTTGTGCAGATCCGGACGCGGAG
GTGATCGCGCTGTCGCCGGGGACGCTCATGGCGACCAACCGGTTCGTGTGCGAGGTGTGCGGCAAGG
GCTTCCAGAGGGACCAGAACCTGCAGCTGCACCGCCGGGGGCACAACCTTCCGTGGCGGCTGCGG
CAGCGCGGCCCCGGGGCGGCGCCGCCGCGCCGGAGGGTCTACGTCTGCCCGGAGCCCGGCTGC
GTGCACCACTCCCCCGCCCGCGCGCTCGGGGACCTCACGGGTATCAAGAAGCACTTTTGCCGCAAG
CACGGCGAGAAGCGATGGGCCTGCCCACGCTGCGGCAAGCGCTACGCCGTCCAGGCCGACCTCAA
GGCCCATGCCAAGACCTGCGGCACCCGCGAGTATCGCTGTGACTGCGGCACGCTCTTCACCAGGTAC
TACTTCCAGCTCGGTTCCCCCCAATTTCGATGGCTCCTTTTTCCTTCCCTTGAGGGACAAATGTTTGTACTT
TTGTTTCATTCATCAGTCGGCGTGCTGGATTGATTCAGGAGAGACAGTTTCGTGACACATCGCGCTTTCTGT
GGCGCTCTCGTCGAGGAGACAGGCAGGGTGCTTGCTGTTCCGACGCCGCCTTCGCCTCGGCCACCTG
ATTTGGAGGAGGTTGAGGAGAATGTAGACAAGGACAAGGAAAAAGAAGAGGAGAATGTGGATAAGCACAA
GGAGAAGGAAGATGAGGAGGGCAAGGGGGGAGAAGATGAAAATGAGACTTCTGCCGTGGCCGAGGTG
GATGAGCCGCAGCACATTGAGGCAACAAGGGAGGAGCCACAGCCACAGCCACAGCGGACTCCGTCG
CCGCCATCTCCAATGCCACAGGAGCAGCACCCAATGGTGGCAATTGTGCCAAATTTGGATGGTATGCTT
CTTTTGCATTTTGCAATGTGTTTGTCTTACTACCTATTCATAGACCATGTTAGATTTATAGCAAACTCATTCTCC
CTAATCCATCTTTATTTCTTATCACTAGCAAAAAAAGGAATGCATCAGTCTTGTTTCTTTCCCAATTTAGCTAG
CCTTATGATCATTCAGTCAACAATGATGAGTGACCTGTTCTTTTGTACCATGCCTATATGTTACAGAGCCAGT
GGTGGTTGTGGAGCCAATTGTGGATATCAAGCAAGAGGAGGAAGATAAGCGAGATGAAGATGTTTGCTTC
CAGGAAGCAGATAATTACGGCGATGCTGAACTAGAAGACTCCAACTTGCCAGATAATGATACCCCGATGC
CTCCTTGTTTCCTCCCATCGCCCTCGGATGCCATTGGTACAGATGGCAGCAGCACCAGTTGTGGCACAG

TCAGCAGCGCATCCAATTCCATCGTGCCAGCAACGACGACTAGCACATTTGCGGGGCTGTTTGCATCAG
CCACAAAAAGCACCACTCCCCAGAGCAGATCGCTGCGTGATCTTATCGGTGTTGATCCCACCTTCTTTTG
CCTTGCAATCGGCACACCATCCTCTCTGTTCCCGCAGACAGATGCAAGCAACCCCAGCACCTTTGCTCC
ACCTCCAGCACCACACATCTCCGCAACTGCGCTCCTGCAGAAGGCCGCTGAGGCTGGAGCTTCGCAA
GCAGGTACATCTTTCTTGAAGGAGTTTGGTCTTGCAAGTTCCTCCTCATCAACCCCATCCAGGCCACCTCA
AGGAAGGTCTATGGATTGCTCAACACAATCCCAACGGCCCCAAGGAAGGTTCGTCGACAGCAGCTCAAT
ACAATCCCGATTACCTCAAGACAGGTTCATCGAGAGCAGCTCAACACAATGCCGGTTACCTCAAGACAG
GTTCATCGAGAGCAGCTCAACACAATCCCGGTTACCTCAAGACAGGTTCATCGACAGCAGTCCAACACA
ATCCCGGTTACCTCAAGACAGGTTCATCGACAGCAGTTCAACACAATTCCGGTTACCTCAAGACAGGTTC
ATCGACAACAGCTCAATACAATCCCGGGTACCTCAAGACAGGTTCATCGACACCAGCTCAACACAATCC
CGGTTACCTCAAGACAGGTTCATCGACAGCAGCTCAACACAATCCCGGTTACCTCAAGACAGGTTCATC
GACAGCAGCTCAACACAATCCCGGTTACCTCAAGACAGGTTCATCGATAACTCGATGCCATCCAAGCTTT
CTCAAGGGAGATTCATGGATACCTCACTGCCATCCCAGCAGCTGCTACCTCAAGGAAGGTTCTTTGACAA
CTCGCCGCCATCAAATCTGTCTCAAGGAAGGTTCTTCGAAAACTCGCAACCATCGAATCCACCTCAAGG
AAGGTTCTTCATCAACTCACCGCCATCTAATCTACCTCATGGAAGGTTCACTGATTACTCAACACCAGGAAT
GTTCATCGATAGCTCGACACTACCCAGGCTGCCTCAAGGAAGGTACATTGATAGCTTGCCACAGTCGAGG
CTACCACAAGGAAGGTACATGGATAACTCACCACCGGCCCAGCTTCCACAGAGAAGGTTGGCTGATAAC
AATCCAGAGCAGTGGCACCAAAGGAGCAATAATCATAACCAGCTAATGGATATGGAGCCTGGGCCGATG
GTATCTGGTAGCCTTGGCCTTGGCCTGGCCTATGAAGGTTCAAATCCAAGGTTGCCAGATTTGATGATGGG
GCAATCACCACTGTTCGGTCCCAAGCCTGCCACTCTGGACTTCCTTGGGCTTGGCATCGGAGGGACCAT
GGGCGGCGGCTCCACGGCGGCCAACGGTGGTGGCCTACCGGCATTGATGGTGGGTGGAGAGCTGGA
CATGGGGTCTGCCGCACAGGCGCCCTCTCCATGGGAGGAGGCACAGAGAAAGACCAACGGCCGCAC
GATCCTGTGA

>SDW3_Hv287_gDNA

ATGCCGCCCAATCCGACGGAGCCGGAGCAGCCGGAGGCGGCCGCGACGCCGGCGCCGCCCAAGA
AGAAGAGGAACCTCCCCGGGACGCCAGGCGAGAGCGCGTGCTTCGGATTTTGCGGGGTTGTTGGTAC
GGCTCGCGCGAGCTTTCTTAGTCTGACGGTGGTCGGTTTCCGTGTGCTTGTGCAGATCCGGACGCGGAG
GTGATCGCGCTGTCGCCGGGGACGCTCATGGCGACCAACCGGTTCGTGTGCGAGGTGTGCGGCAAGG
GCTTCCAGAGGGACCAGAACCTGCAGCTGCACCGCCGGGGGCACAACCTTCCGTGGCGGCTGCGG
CAGCGCGGCCCCGGGGCGGCGCCGCCGCGCCGGAGGGTCTACGTCTGCCCGGAGCCCGGCTGC
GTGCACCACTCCCCCGCCCGCGCGCTCGGGGACCTCACGGGTATCAAGAAGCACTTTTGCCGCAAG
CACGGCGAGAAGCGATGGGCCTGCCCACGCTGCGGCAAGCGCTACGCCGTCCAGGCCGACCTCAA
GGCCCATGCCAAGACCTGCGGCACCCGCGAGTATCGCTGTGACTGCGGCACGCTCTTCACCAGGTAC
TACTTCCAGCTCGGTTCCCCCCAATTTCGATGGCTCCTTTTTCCTTCCCTTGAGGGACAAATGTTTGTACTT
TTGTTTCATTCATCAGTCGGCGTGCTGGATTGATTCAGGAGAGACAGTTTCGTGACACATCGCGCTTTCTGT
GGCGCTCTCGTCGAGGAGACAGGCAGGGTGCTTGCTGTTCCGACGCCGCCTTCGCCTCGGCCACCTG
ATTTGGAGGAGGTTGAGGAGAATGTAGACAAGGACAAGGAAAAAGAAGAGGAGAATGTGGATAAGCACAA
GGAGAAGGAAGATGAGGAGGGCAAGGGGGGAGACGATGAAAATGAGACTTCTGCCGTGGCCGAGGTG
GATGAGCCGCAGCACATTGAGGCAACAAGGGAGGAGCCACAGCCACAGCCACAGCGGACTCCGTCG
CCGCCATCTCCAATGCCATAGGAGCAGCACCCAATGGTGGCAATTGTGCCAAATTTGGATGGTATGCTTC
TTTTGCATTTTGCAATGTGTTTGTCTTACTACCTATTCATAGACCATGTTAGATTTATAGCAAACTCATTCTCCCT
AATCCATCTTTATTTCTTATCACTAGCAAAAAAAGGAATGCATCAGTCTTGTTTCTTTCCCAATTTAGCTAGCC
TTATGATCATTCAGTCAACAATGATGAGTGACCTGTTCTTTTGTACCATGCCTATATGTTACAGAGCCAGTGGT

GGTTGTGGAGCCAATTGTGGATATCAAGCAAGAGGAGGAAGATAAGCGAGATGAAGATGTTTGCTTCCAG
GAAGCAGATAATTACGGCGATGCTGAACTAGAAGACTCCAACTTGCCAGATAATGATACCCCGATGCCTC
CTTGTTTCCTCCCATCGCCCTCGGATGCCATTGGTACAGATGGCAGCAGCACCAGTTGTGGCACAGTCA
GCAGCGCATCCAATTCCATCGTGCCAGCAACGACGACTAGCACATTTGCGGGGCTGTTTGCATCAGCCA
CAAAAAGCACCACTCCCCAGAGCAGATCGCTGCGTGATCTTATCGGTGTTGATCCCACCTTCCTTTGCCT
TGCAATCGGCACACCATCCTCTCTGTTCCCGCAGACAGATGCAAGCAACCCCAGCACCTTTGCTCCAC
CTCCAGCACCACACATCTCCGCAACTGCGCTCCTGCAGAAGGCCGCTGAGGCTGGAGCTTCGCAAGC
AGGTACATCTTTCTTGAAGGAGTTTGGTCTTGCAAGTTCCTCCTCATCAACCCCATCCAGGCCACCTCAAG
GAAGGTCTATGGATTGCTCAACACAATCCCAACGGCCCCAAGGAAGGTTCGTCGACAGCAGCTCAATAC
AATCCCGATTACCTCAAGACAGGTTCATCGAGAGCAGCTCAACACAATGCCGGTTACCTCAAGACAGGTT
CATCGAGAGCAGCTCAACACAATCCCGGTTACCTCAAGACAGGTTCATCGACAGCAGTCCAACACAATC
CCGGTTACCTCAAGACAGGTTCATCGACAGCAGTTCAACACAATTCCGGTTACCTCAAGACAGGTTCATC
GACAACAGCTCAATACAATCCCGGGTACCTCAAGACAGGTTCATCGACACCAGCTCAACACAATCCCGG
TTACCTCAAGACAGGTTCATCGACAGCAGCTCAACACAATCCCGGTTACCTCAAGACAGGTTCATCGACA
GCAGCTCAACACAATCCCGGTTACCTCAAGACAGGTTCATCGACAGCAGCTCAACACAATCCCGGTTAC
CTCAAGACAGGTTCATCAATAACTCGATGCCATCCAAGCTTTCTCAAGGGAGATTCATGGATACCTCACTG
CCATCCCAGCAGCTGCTACCTCAAGGAAGGTTCTTTGACAACTCGCCGCCATCAAATCTGTCTCAAGGA
AGGTTCTTCGAAAACTCGCAACCATCGAATCCACCTCAAGGAAGGTTCTTCATCAACTCACCGCCATCTA
ATCTACCTCATGGAAGGTTCACTGATTACTCAACACCAGGAATGTTCATCGATAGCTCGACACTACCCAGG
CTGCCTCAAGGAAGGTACATTGATAGCTTGCCACAGTCGAGGCTACCACAAGGAAGGTACATGGATAACT
CACCACCGGCCCAGCTTCCACAGAGAAGGTTGGCTGATAACAATCCAGAGCAGTGGCACCAAAGGAG
CAATAATCATAACCAGCTAATGGATATGGAGCCTGGGCCGATGGTATCTGGTAGCCTTGGCCTTGGCCTG
GCCTATGAAGGTTCAAATCCAAGGTTGCCAGATTTGATGATGGGGCAATCACCACTGTTCGGTCCCAAGC
CTGCCACTCTGGACTTCCTTGGGCTTGGCATCGGAGGGACCATGGGCGGCGGCTCCACTGCGGCCAA
CGGTGGTGGCCTACCGGCATTGATGGTGGGTGGAGAGCTGGACATGGGGTCTGCCGCACAGGCGCCC
TCTCCATGGGAGGAGGCACAGAGAAAGACCAACGGCCGCACGATCCTGTGA
